# Comprehensive analyses indicated the association between m6A related long non‐coding RNAs and various pathways in glioma

**DOI:** 10.1002/cam4.4913

**Published:** 2022-06-06

**Authors:** Zhuohui Chen, Wei Zhang, Zhouyi Yan, Mengqi Zhang

**Affiliations:** ^1^ Department of Neurology, Xiangya Hospital Central South University Changsha China; ^2^ Department of Neurosurgery, Xiangya Hospital Central South University Changsha China; ^3^ National Clinical Research Center for Geriatric Disorders, Xiangya Hospital Central South University Changsha China

**Keywords:** glioma, immunotherapy sensitivity, m6A related lncRNA, prognosis prediction, tumor microenvironment, tumor mutation burden

## Abstract

**Background:**

Glioma is one of the most malignant brain tumors and diseases. N6‐methyladenosine modification (m6A) is the most abundant and prevalent internal chemical modification of mRNA and long non‐coding RNAs (lncRNAs) in eukaryotes. Nevertheless, the correlated pathways and clinical utilization of m6A‐related lncRNAs have not been fully evaluated in glioma.

**Methods:**

Public RNA‐sequencing and clinical annotation data were retrieved from TCGA, CGGA and GEO database. Differential expression analysis and univariate Cox regression analysis were performed to identify the m6A‐related and differentially expressed lncRNAs with prognostic function (m6A‐DELPF). The consensus clustering was performed to identify the expression pattern of m6A‐DELPF. LASSO Cox regression analysis was performed to construct the lncRNA‐based signature. The CIBERSORT and ESTIMATE algorithms were performed to analyze immune infiltration and tumor microenvironment, respectively. Immunotherapy sensitivity analysis was performed using data from TCIA. The small molecule drugs prediction analysis was performed using The Connectivity Map (CMap) database and STITCH database. A competing endogenous RNAs (ceRNA) network was constructed based on miRcode, miRDB, miRTarBase, TargetScan database.

**Results:**

Two clusters (cluster1 and cluster2) were identified after unsupervised cluster analysis based on m6A‐DELPF. Additionally, a 15‐gene prognostic signature namely m6A‐DELPFS was constructed. Analyses of epithelial‐mesenchymal‐transition score, tumor microenvironment, immune infiltration, clinical characterization analysis, and putative drug prediction were performed to confirm the clinical utility and efficacy of m6A‐DELPFS. The potential mechanisms including tumor immune microenvironment of m6A‐DELPF influence the initiation and progression of glioma. A clinically accessible nomogram was also constructed based on the m6A‐DELPF and other survival‐relevant clinical parameters. Two miRNAs and 114 mRNAs were identified as the downstream of seven m6A‐related lncRNAs in a ceRNA network.

**Conclusion:**

Our present research confirmed the clinical value of m6A related lncRNAs and their high correlation with tumor immunity, tumor microenvironment, tumor mutation burden and drug sensitivity in glioma.

## Graphical abstract

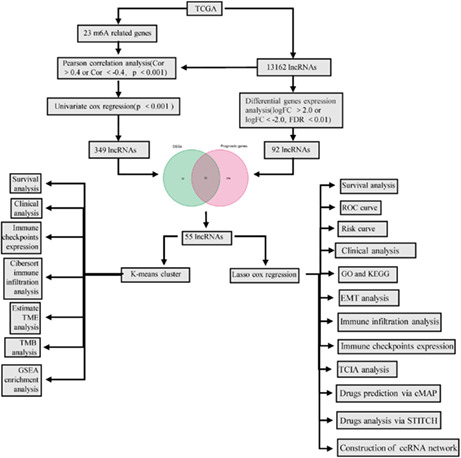

## 1 INTRODUCTION

Gliomas are the most common types of tumors in the nervous system, including diffuse and definitive types.^1^ As the second leading cause of death in the central nervous system, glioma is also one of the most malignant tumors of the brain and diseases, with a high incidence and low survival rate.^2^ Gliomas were classified numerically into (I‐IV) according to The World Health Organization (WHO) based on the pathological characteristics of malignant tumors.^3^

The main reason for the poor prognosis of glioma is that the current treatment methods (including surgical resection, radiotherapy and chemotherapy, etc.) are not satisfactory and the efficacy is not ideal, which is mainly caused by the chemical and radiation resistance of tumor stem cells(TSCs). It has been described that many genetic mutations can increase the risk of gliomas such as *NF1*/ *2*, *TSC1*/ *2*, *TP53*, *PTEN*, *APC*, *IDH1/2*, *hMLH1/2*, and *PMS2*.^1^, ^4^ The clinical treatment of glioma is mainly determined by not only the size, type, grade, and location of the tumor, but also the patient's age and overall health. Therefore, it is extremely urgent to find the prediction and treatment targets of different grades of glioma.

N6‐methyladenosine modification (m6A) is an evolutionarily conserved RNA modification and refers to the addition of a methyl group to the adenosine N6 site.^5^, ^6^ It is considered one of the most substantial and prevalent internal modifications of RNAs in eukaryotes. The formation of m6A is a reversible dynamic process, consisting of “writers” with methyltransferase activity, “erasers” with demethylase activity, and “readers” with effector activity.^7^ “Writers” are composed of methyltransferase‐like 3 (METTL3), methyltransferase‐like 14 (METTL14) and Wilms tumor 1 related protein (WTAP). The m6A modification can regulate the fate of mRNA to influence its metabolism like splicing, and decay.^8^, ^9^ In addition, m6A modification is also closely related to immunity. The m6A modification regulates T cell activation, proliferation, differentiation, metabolic reprogramming and other physiological processes by regulating the mRNA degradation of suppressor of cytokine signaling (SOCS) family genes, and it also regulates the maturation and function of dendritic cells by affecting the translation process of costimulatory molecules.^10^, ^11^ The m6A is also involved in multiple processes such as the maintenance of cell pluripotency, embryonic development and circadian rhythm maintenance.^12^, ^13^, ^14^ Notably, a lot of researches have demonstrated that abnormal changes in m6A also affect glioma activity, including proliferation and metastasis.^15^, ^16^, ^17^, ^18^ For example, down‐regulation of METTL3 or METTL14 can enhance the malignancy of tumors and promote tumor proliferation and metastasis.^16^

Non‐coding RNAs whose length is >200 nucleotides (nt) are generally considered long non‐coding RNAs (lncRNAs).^19^ Recent evidence indicated that the dysregulated lncRNA is highly relevant to the proliferation, growth, metastasis and prognosis of glioma.^20^, ^21^, ^22^ HULC LncRNA can regulate ESM‐1 through the PI3K/Akt/mTOR signaling pathway, thereby promoting angiogenesis in human gliomas and increasing tumor invasion and metastasis.^23^ In summary, understanding whether and how m6A‐related lncRNAs are correlated with glioma is helpful to identify potential therapeutic targets.

In multiple glioma datasets, there is still a lack of comprehensive and detailed researches on the utilization of m6A‐related lncRNAs. We attempted to fill this gap with adequate demonstration to determine the efficacy of the signature and the possible downstream mechanisms of this special lncRNA subgroup. Herein, based on The Cancer Genome Atlas (TCGA) lower grade glioma (LGG) and glioblastoma multiforme (GBM) datasets, we performed bioinformatics and statistical analysis on the information of glioma patients, identified 55 m6A‐related and differentially expressed lncRNAs with prognostic function (m6A‐DELPF) and then comprehensively dug into the clinical value and potential downstream mechanisms of this special lncRNA subgroup in glioma. These results were partially confirmed in several external datasets including CGGA, GSE108474 and GSE16011.

## 2 MATERIALS AND METHODS

### 2.1 Data collection

**FIGURE 1:**
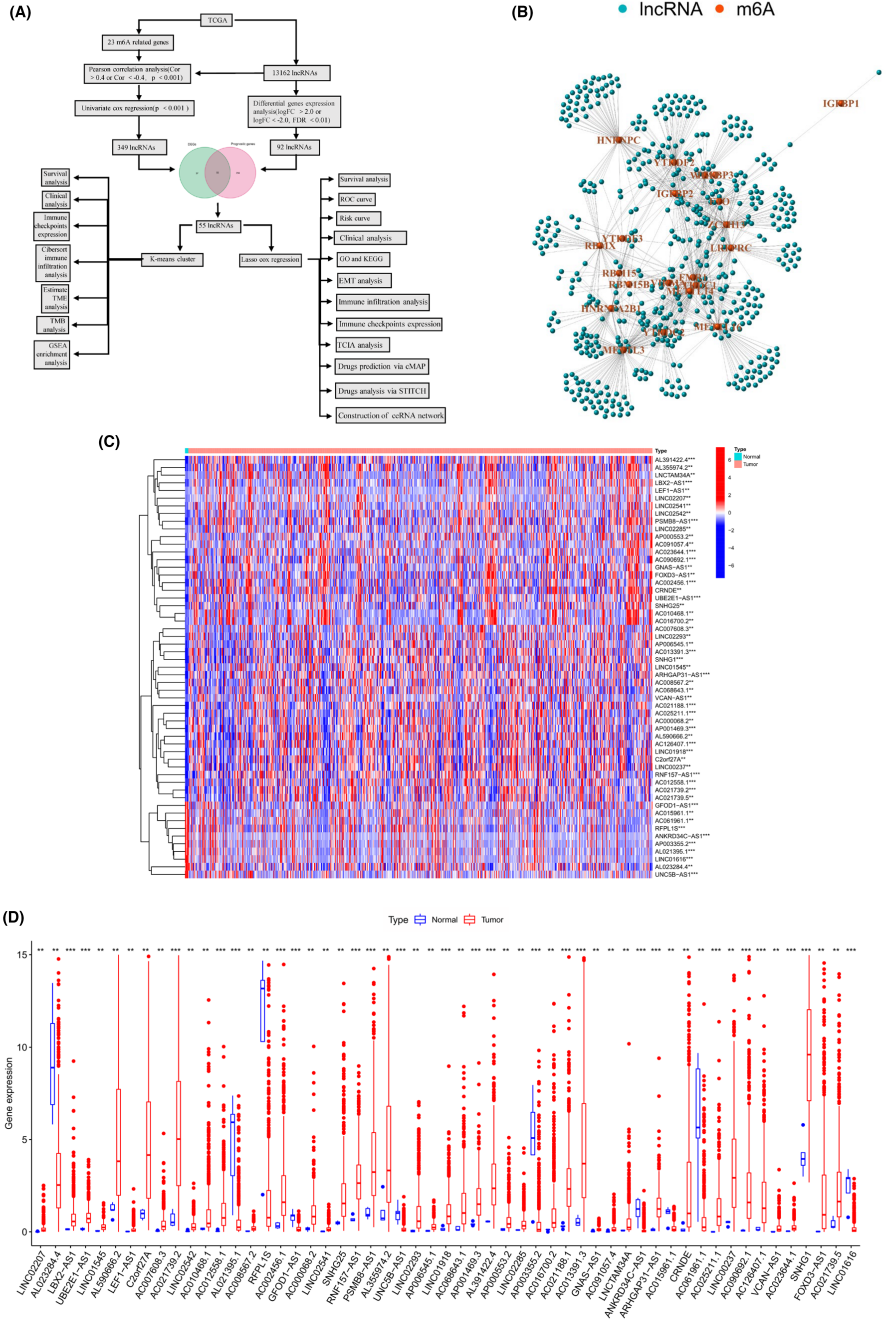
The analysis flow chart and the identification of m6A‐related lncRNAs or lncRNAs differently expressed in patients with glioma and healthy people. (A) The analysis flow chart. (B) The network of the correlation between the lncRNAs and the m6A‐related genes through Pearson correlation analysis (|R| >0.4, *p* < 0.001). The nodes represent lncRNA or m6A‐related genes(The name of 23 m6A‐related genes had been annotated.). (C) The heatmap of different expressions about the 55 m6A‐DELPF in patients with glioma and normal people. *p < 0.05, **p < 0.01 and ***p < 0.001. (D) The boxplot of different gene expressions about the 55 m6A‐DELPF between the patients with glioma and the healthy people. *p < 0.05, **p < 0.01 and ***p < 0.001

The flow chart of our current study was shown in Figure 1A. Public RNA‐sequencing FPKM data and clinical annotations were retrieved from The Cancer Genome Atlas (TCGA), Chinese Glioma Genome Atlas (CGGA) and Gene Expression Omnibus (GEO) database. mRNA expression, lncRNA expression, clinical data of TCGA‐LGG, TCGA‐GBM, CGGA, GSE108474 and GSE16011 were collected in https://portal.gdc.cancer.gov/, http://www.cgga.org.cn/ and https://www.ncbi.nlm.nih.gov/geo/.

### 2.2 Identification of m6A‐related lncRNAs

To identify m6A related genes, we firstly confirmed the expression mode of 23 m6A related genes including m6A writers (METTL3, METTL14, METTL16, WTAP, VIRMA, ZC3H13, RBM15, RBM15B), readers (YTHDC1, YTHDC2, YTHDF1, YTHDF2, YTHDF3, HNRNPC, FMR1, LRPPRC, HNRNPA2B1, IGFBP1, IGFBP2, IGFBP3, RBMX) and erasers (FTO, ALKBH5).^24^, ^25^, ^26^, ^27^, ^28^ Then, we utilized Pearson's correlation to screen out the lncRNAs shared a similar expression mode to these 23 m6A related genes. LncRNAs with |R| >0.4 and *p* < 0.001 were considered as m6A‐related lncRNAs.^29^ The m6A‐related LncRNA and its correlation coefficient obtained by coefficient correlation analysis were listed in Table S1. Afterward, differential expression analysis was conducted using Limma R package^30^ version 3.44.3 to compare the expression of m6A‐related lncRNAs between tumor (*n* = 698) and normal (*n* = 5) tissues. The expression threshold was set as |log FC| > 2 and the p value threshold was set as 0.05 (adjusted by false discovery rate [FDR]). Differentially expressed genes (DEGs) were listed in Table S3. Additionally, a univariate Cox regression analysis with a p filter of 0.001 was also performed to identify the clinical‐relevant m6A‐related lncRNAs (listed in Table S4). Finally, we intersected the lncRNAs from the above two analyses to identify the m6A‐related and differentially expressed lncRNAs with prognostic function (m6A‐DELPF) (listed in Table S5, S6).

### 2.3 Clustering expression pattern identification

An unsupervised clustering analysis (k‐means clustering) was performed in TCGA cohorts using ConsensusClusterPlus^31^ R package version 1.52.0 with 500 repetitions to guarantee the stability of classification. The analysis clustered the patients from TCGA into cluster1 and cluster2. The clustering results of each patient from TCGA cohorts were listed in Table S7.

### 2.4 Gene enrichment analysis

To determine the possible pathways involved by the m6A‐related lncRNAs, we performed gene set enrichment analysis (GSEA) by using GSEA software version 4.1.0^32^ (downloaded from http://www.broadinstitute.org/gsea/). The gene set “h.all.v7.4.symbols.gmt” for GSEA analysis was obtained from the MSigDB database^33^ for the identification of different hallmark pathways between cluster1 and cluster2. The enriched pathways of cluster1 and cluster2 were listed in Table S8, S9, respectively.

Gene ontology (GO) and Kyoto Encyclopedia of Genes and Genomes (KEGG) pathway analysis were also executed to annotate differential expressed genes between the two different risk groups by utilizing ClusterProfiler R package version 3.16.1^34^ (listed in Table S16, 17).

### 2.5 The construction and validation of m6A‐DELPF signature

To construct the lncRNA based signature, we obtained the training group and the testing group via randomly dividing all sample from TCGA cohorts into two groups. The randomization was confirmed by the chi‐square test in Table S11. Then, we utilized the data of the training group and performed the least absolute shrinkage and selection operator (LASSO) Cox regression analysis utilized in Glmnet^35^ R package version 4.0–2 to construct an m6A‐DELPF signature (m6A‐DELPFS) based on the data of training group. Afterward, the risk score for each patient can be defined and calculated as the following formula:

(1) $$\text{Risk Score}=\sum_{i=1}^{n}\left(\mathrm{Coe}f\left(X_{i}\right)*\mathrm{Exp}\left(X_{i}\right)\right)$$

$\text{Coef}\left(X_{i}\right)$ and $\mathrm{Exp}\left(X_{i}\right)$ indicated the coefficient of each m6A‐DELPF$\;\left(X_{i}\right)$ and the expression levels of the above genes. All patients can be divided into low‐risk and high‐risk based on the calculated risk score and relevant median risk score(listed in Table S12). Receiver operating characteristic curve (ROC) analysis, clinical independence analysis, stratified survival analysis, etc. were used to validate the efficacy of m6A‐DELPFS from different aspects.

### 2.6 The calculation of tumor microenvironment (TME) immune cell infiltration abundance

The CIBERSORT^36^ algorithm (https://cibersort.stanford.edu) was utilized to assess the relative abundance of 22 immune cells by using support vector regression to deconvolute the bulk RNA‐seq data from each patient in TCGA cohorts. The input of reference gene sets for 22 immune cells was captured from the publication of Newman et al.^37^; a 100‐time permutation test was also performed as the default parameters of the CIBERSORT algorithm.

### 2.7 The calculation of admixture of stromal and immune cell to predict tumor purity

The ESTIMATE^38^ algorithm was performed in each patient in TCGA cohorts to calculate the scores of stromal and immune cells based on ssGSEA analysis. The input of specific gene sets of stromal and immune cells was collected from the publication of Yoshihara et al. The higher the predicted score is, the more that kind of cells is in the tumor tissues. In addition, the ESTIMATE algorithm can directly calculate the purity scores of the tumor sample since the ESTIMATE Score is negatively correlated with tumor purity. A lower score of ESTIMATE represents a purer tumor tissue, that is, less infiltration of stromal and immune cells into the tumor tissue.

### 2.8 The calculation of epithelial‐mesenchymal transition (EMT) score

By utilizing the gene sets including 25 epithelial (E) marker genes and 52 mesenchymal (M) marker genes constructed by Mak et al.,^39^ we defined the EMT score of each sample using the following formula:

(2) $$\mathrm{EMT}\;\text{Score}=\sum_{i}^{N}\frac{M^{i}}{N}-\sum_{j}^{n}\frac{E^{j}}{n}$$

In formula (2), N and n represented the number of mesenchymal marker genes and epithelial marker genes, respectively. Mi and Ej represented the expression level of specific mesenchymal marker gene i and epithelial marker genes j, respectively. In short, EMT was more likely to occur in patients with higher EMT Score calculated by the formula (2). The EMT score calculated for each patient sample was listed in Table S19.

### 2.9 The calculation of tumor mutation burden (TMB)

By using the somatic mutation data from TCGA cohorts, we calculated tumor mutation burden (TMB) in a tumor sample as the overall number of replacement, insertion, and deletion mutations per megabase (MB) in the exon coding region of the evaluated gene.^40^ The TMB calculated for each patient sample was listed in Table S20.

### 2.10 Construction of ceRNA network

On the basis of the aberrantly expressed m6A related lncRNA (*n* = 7), miRNA (*n* = 2), and mRNA (*n* = 114) which can be identified in the following database between low‐ and high‐risk patients, the ceRNA network was constructed. The direct lncRNA‐miRNA interactions were predicted in miRcode^41^ (http://www.mircode.org/). The direct miRNA‐mRNA interactions were predicted by using the combined results of miRDB^42^ (http://mirdb.org/), miRTarBase^43^ (http://miRTarBase.cuhk.edu.cn/) and TargetScan^44^ (http://www.targetscan.org/vert_72/). mRNAs with at least two positive results reported by the above three databases were considered valid. The ceRNA network was obtained in Cytoscape v3.8.1. The exact link relationship was listed in Table S24.

### 2.11 The immunotherapy sensitivity analysis based on m6A‐DELPFS

The immunotherapy sensitivity data were downloaded from The Cancer Immunome Atlas (TCIA, https://tcia.at/home). TCIA retrieved the comprehensive immunogenomic analyses of RNA‐seq data for 20 solid cancers from TCGA and publications of Van Allen E.M. et al and Hugo W. et al.^45^, ^46^ The data of TCGA‐GBM cohort were utilized to discover the relationship between immunotherapy sensitivity and m6A‐DELPFS in our present study. The used TCIA data were listed in Table S21.

### 2.12 The identification of potential small molecule drugs and downstream target proteins

On the basis of the DEGs between low‐ and high‐risk groups, the small molecule drug prediction analysis was performed via utilizing The Connectivity Map (CMap) database^47^version build 02 (https://portals.broadinstitute.org/cmap/). The enrichment scores of each drug ranging from −1 to 1 were predicted as the results of the analysis. The negative score indicates the reversal effect of the drug on the input gene set, so suggesting the antitumor ability of the drug on the tumor‐associated gene set. Thus, the closer the enrichment score of a drug is to −1, the more likely the drug is to be anti‐tumor. The small molecule compounds were then chosen with the filter of enrichment scores <−0.7 and *p* < 0.05. The information of the top 20 prospective drugs with the significant score for glioma treatment was listed in Table S22. Afterward, to identify the putative targets of these predicted drugs, the STITCH^48^database version 5.0 (http://stitch.embl.de/) was used to predict the target proteins. The STITCH database is a platform to predict drug–drug, drug‐protein and protein–protein interactions based on current publications and other predicting databases. The selected small molecule drugs were sent to perform the analysis to target the common target protein of these drugs. Afterward, the predicted target proteins were annotated using GO and KEGG analyses which have been mentioned above (see Table S23).

### 2.13 Statistical analysis

All the statistics were analyzed using the R software version 4.0.3 (https://www.R‐project.org/). Spearman's rank correlation analysis was performed to assess the linear relevance between risk score and the expression of immune checkpoint‐related genes. Survival curves between different groups were obtained via using Kaplan–Meier analysis. Univariate and multivariate Cox analyses were performed to confirm the clinical independence of m6A‐DELPF signature from other clinical parameters. Based on the results of multivariate Cox analysis conducted in the whole TCGA dataset, a nomogram was constructed by using rms R package version 6.1.0 and thereafter assessed by the concordance index (C‐index) and the calibration curve with a bootstrap resampling of 1000. The closer the value of the C‐index is to 1, the better the performance of the nomogram is. The decision curve analysis (DCA) was also performed on the training set, testing set and the whole TCGA datasets with a clinical impact curve (CIC) constructed to determine the net benefit of the nomogram using rmda R package version 1.6.^49^ Kruskal‐Wallis H‐test was used to compare the difference between groups. All statistical analysis was two‐sided. The *p* < 0.05 was considered statistically significant.

## 3 RESULTS

### 3.1 Identification of m6A‐DELPF in TCGA

In order to obtain the original data that can be used, we firstly extracted, screened and identified 13,162 lncRNAs and 23 m6A‐related gene expression matrices from the downloaded TCGA database. The working flow chart demonstrating a series of subsequent analyses was shown in Figure 1A. Herein, we defined what m6A‐related LncRNA is. As long as the expression value of the selected lncRNA is related to the expression of one or more of the 23 identified m6A‐related genes (| Pearson R | > 0.4, *p* < 0.001), it can be identified as m6A‐related lncRNA. For this, we performed Pearson correlation analysis, and got 1119 lncRNAs that are significantly related to m6A. In order to correlate the obtained m6A‐related lncRNAs better with the prognosis of glioma patients, we performed a univariate cox regression on the 1119 m6A‐related lncRNAs, and obtained 349 m6A‐related lncRNAs with prognostic function (m6A‐LPF) (*p* < 0.001). Then, we also performed differential gene expression analysis on the 13,162 lncRNAs, and obtained 92 lncRNAs with significant differential expression between glioma and normal tissues (|logFC| > 2.0, FDR <0.01). Subsequently, we intersected 349 m6A‐LPF and 92 differentially expressed genes, and obtained 55 differentially expressed m6A‐LPF, namely m6A‐DELPF. The correlations between 1119 lncRNAs in the TCGA data set and m6A‐related genes were shown in Table S1. To intuitively and vividly display the correlation between the lncRNAs and the m6A‐related genes, the network of the results in Pearson correlation analysis was visualized in Figure 1B. The results of differential expression of these 55 m6A‐DELPF between glioma patients and normal controls are shown in Figure 1C,D. Group information of samples about the intersection genes in survival TCGA‐LGG/GBM is shown in Table S6.

### 3.2 Distinct patterns of m6A‐DELPF expression associated with clinicopathological characteristics

**FIGURE 2:**
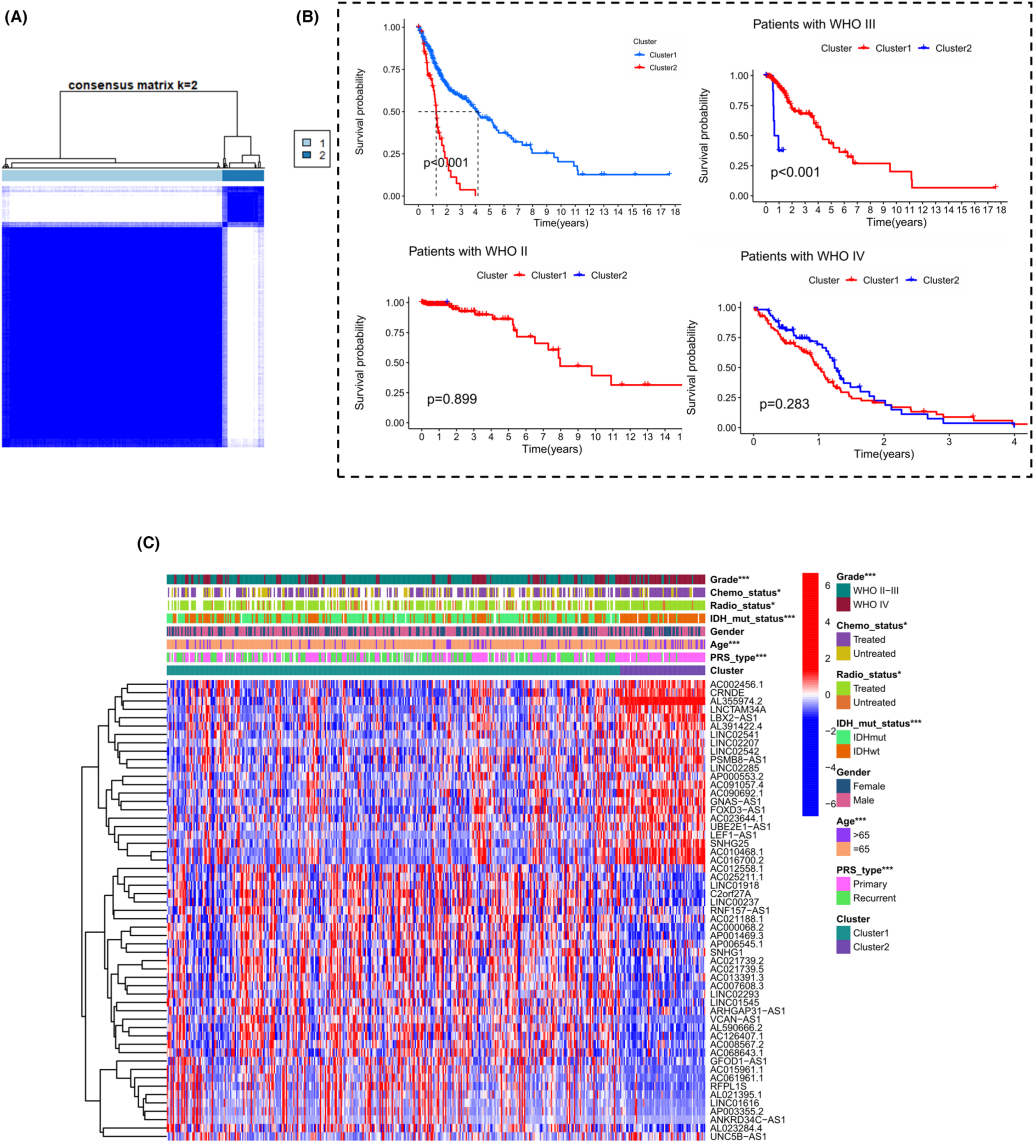
The patterns of m6A‐DELPF expression and the biological characteristics of each pattern. (A) The consensus matrix of the clustering analysis of the 443 samples based on m6A‐DELPF expression profiles via k‐means clustering (k = 2). (B) Kaplan–Meier curves show the overall survival of patients in cluster1 or cluster2 in m6A‐DELPF expression in the TCGA cohort. In the two‐sided log‐rank test, *p* < 0.05 is considered statistically significant. There was no significantly different between the survival prognosis of the cluster 1 and cluster 2 in patients with WHO grade II and IV. And for the patients with WHO grade III, the overall survival of cluster 1 was better than that of cluster 2. (C) Heatmap visualizing the gene expression of the 55 m6A‐DELPF in distinct m6A‐DELPF expression patterns and displaying associations between the expression levels of the 55 lncRNAs and clinicopathological features in the TCGA cohort. *p < 0.05, **p < 0.01 and ***p < 0.001

In order to better classify glioma patients with different properties and distribution of lncRNA expression patterns, we performed k‐means clustering on 443 glioma patients based on the selected 55 lncRNA expression profiles. The clustering results showed that 373 patients were identified as cluster1, while the remaining 70 patients were identified as cluster2 (Figure 2A). In the prognostic analysis of the expression pattern of m6A‐DELPF, the expression pattern in subtype cluster1 showed a particularly significant survival advantage (Figure 2B; *p* < 0.001). In further clinical stratification analysis, a significantly different survival rate was observed between cluster 1 and cluster 2 in grade III but not in grade II and IV patients (Figure 2B). Subsequently, to determine whether this clustering method was applicable to populations with different clinicopathological characteristics, we conducted a hierarchical analysis of m6A‐DELPF based on this classification result and a chi‐square test to determine if different clinical patterns existed between the two clusters. The results showed that this classification had significantly different clinical patterns in WHO grade (*p* = 5.96e‐21), chemotherapy treatment status (*p* = 0.0102), radiotherapy treatment status (*p* = 0.0236), IDH mutation status (*p* = 1.387e‐15), age (*p* = 2.66e‐05), and primary or recurrent glioma (*p* = 6.19e‐10). In short, patients in cluster1 had a higher proportion of WHO II‐III, untreated by chemotherapy and radiotherapy, mutant IDH, age ≤ 65 and primary tumor. However, no significantly different pattern was shown in gender between patients of cluster1 and cluster2 (*p* = 0.521) (Figure 2C, Table S7).

### 3.3 Pathway enrichment analysis via GSEA in cluster1 and cluster2

**FIGURE 3:**
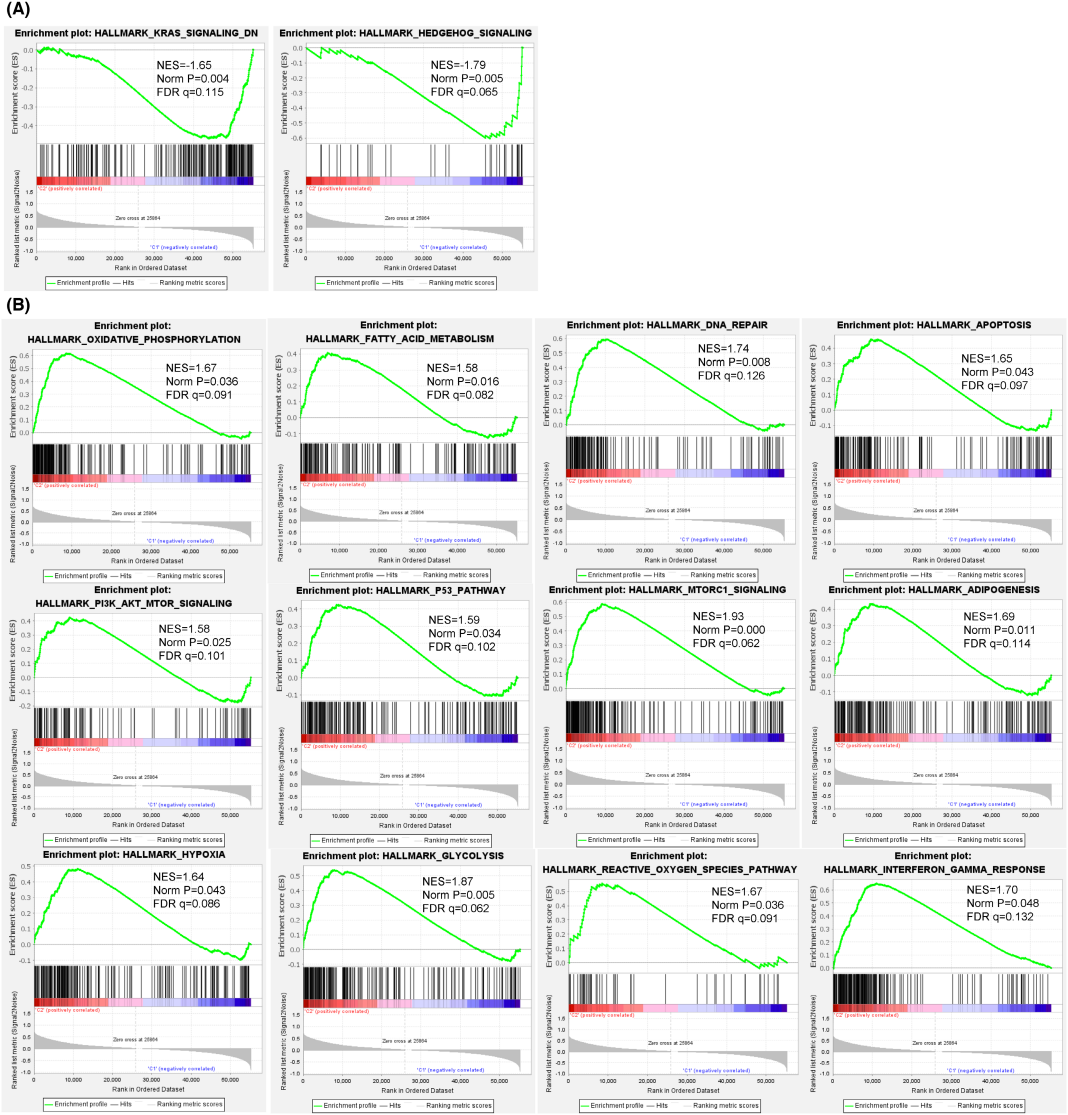
Pathway enrichment analysis between cluster1 and cluster2 via GSEA. (A) Two gene sets including HEDGEHOG SIGNALING and KRAS SIGNALING DN were significantly enriched in cluster1 at nominal *p* value <0.05. (B) 12 of 15 gene sets were significantly enriched in cluster2 at nominal *p* value <0.05, including oxidative phosphorylation, mTORC1 signaling and glycolysis, etc.

In order to further understand the potential molecular biological pathway differences between the Cluster1 and Cluster2, which were divided from the 443 samples based on m6A‐DELPF expression, we analyzed 29,405 genes in Cluster1 and 25,864 genes in Cluster2 by GSEA pathway enrichment. The obtained analysis results showed that 14 gene sets in cluster1 were up‐regulated (Table S8). Moreover, two gene sets including HEDGEHOG SIGNALING (NES = ‐1.79, Norm *p* = 0.005, FDR q = 0.065) and KRAS SIGNALING DN (NES = −1.65, Norm *p* = 0.004, FDR q = 0.115) were significantly enriched (Figure 3A). In cluster2, 36 gene sets were upregulated and 15 gene sets were significantly enriched at nominal *p* value <5%, which mainly included oxidative phosphorylation, mTORC1 signaling, glycolysis, DNA repair, adipogenesis, apoptosis, reactive oxygen species path, p53 pathway, PI3K/AKT/mTOR signaling, fatty acid metabolism and interferon gamma response, etc. (Figure 3B, Table S9). Multiple tumor markers in cluster2 are up‐regulated, with poor prognosis. The results of enrichment of these pathways may enrich our knowledge of the possible cellular and molecular biological mechanisms that contribute to the differences in tumor prognosis between Cluster1 and Cluster2.

### 3.4 Distinct patterns of m6A‐DELPF expression associated with immune checkpoints and immune infiltration

**FIGURE 4:**
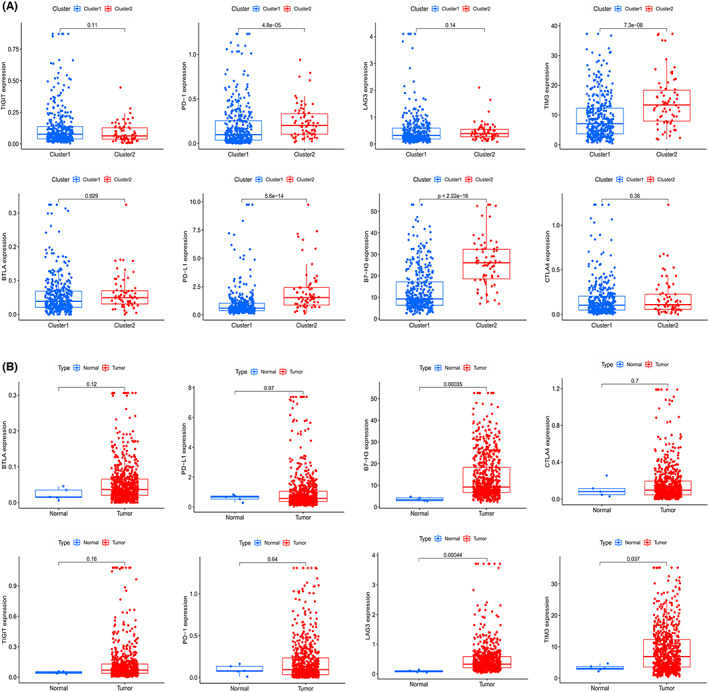
Different expression of immune checkpoint genes between cluster1 and cluster2 or between normal and tumor tissues. (A) The boxplots showing different expressions of TIGHT, PD‐1, LAG3, TIM3, BTLA, PD‐L1, B7‐H3 and CTLA4 between cluster1 (blue) and cluster2 (red). (B) The boxplots displaying differently expressed TIGHT, PD‐1, LAG3, TIM3, BTLA, PD‐L1, B7‐H3 and CTLA4 in patients with glioma (red) and healthy people(blue). *p* < 0.05 is considered statistically significant

Numerous studies have demonstrated the association between TME infiltration of immune cells and the expression of lncRNAs.^50^, ^51^, ^52^ And the pathway enrichment analysis via GSEA uncovered the potential mechanism of difference between cluster1 and cluster2, such as p53 pathway, PI3K/AKT/mTOR signaling, interferon gamma response, etc. Interferon γ is an important cytokine in the body, which can coordinate the immune response by regulating the transcription of immune‐related genes.^53^ After further analysis, it is not difficult to find that the genes in cluster1 and cluster2 are mostly enriched in immune‐related genes. Therefore, we attempted to evaluate the relationship between m6A‐DELPF and immune checkpoints in TME. We analyzed the expression of immunosuppression‐related genes in Cluster1 and Cluster2, and found that compared to cluster1, a variety of immunosuppressive‐related genes were up‐regulated in cluster2, such as PD‐L1, B7‐H3, BTLA, PD‐1, and TIM3 (Figure 4A). However, we also observed that some immunosuppression‐related genes, such as LAG3, TIGIT and CTLA4, did not show significant differences in Cluster1 and Cluster2. Then, in order to confirm whether these immunosuppression‐related genes are indeed involved in the occurrence and development of glioma, we analyzed the expression of immunosuppression‐related genes in glioma patients and normal people. We found that in patients with glioma, B7‐H3, LAG3 and TIM3 were significantly raised, while the expression of PD‐L1, BTLA, TIGIT, PD‐1 and CTLA4 did not seem to be significantly different from those of normal individuals (Figure 4B). Those suggested that not all immunosuppression‐related genes were up‐regulated during the occurrence and development of glioma, but only part of the genes may be expressed abnormally. For instance, B7‐H3, TIM3 and LAG3 were more likely to associate with the occurrence of glioma, while B7‐H3, TIM3, BTLA, PD‐1, and PD‐L1 were more likely to associate with the development of glioma via immunosuppression.

**FIGURE 5:**
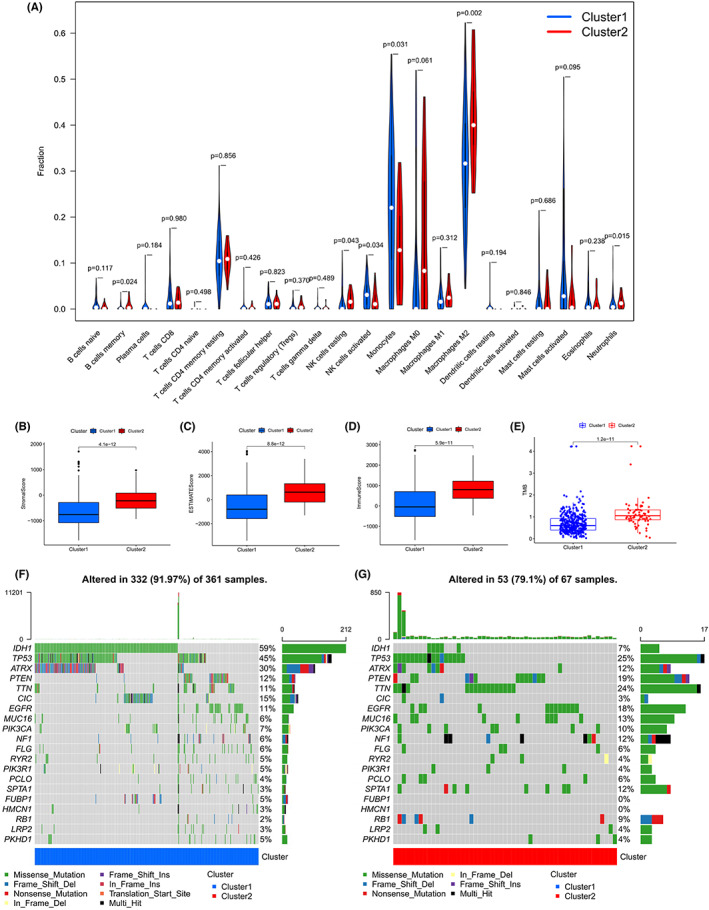
The analysis of immune infiltration via CIBERSORT and the tumor microenvironment via ESTIMATE. (A) Different distribution of immune infiltrating cells in cluster 1 and cluster 2. *p* < 0.05 is considered statistically significant. (B‐E) the boxplots respectively shows the different stromal score, ESTIMATE score, immune score and TMB between cluster1 and cluster2. *p* < 0.05 is considered statistically significant. (F–G) the oncoplot shows the comprehensive mutation status of the genes with the top 20 mutation number in cluster1 and cluster2, respectively

In order to compare the TME immune‐infiltrating cells between the two different m6A‐DELPF expression patterns cluster1 and cluster2, we adopted the CIBERSORT method. The results of this approach indicated that the expression pattern of m6A‐DELPF was closely related to the infiltration of immune cells in the TME (Figure 5A, Table S10). For example, through comparing two modes of m6A‐DELPF expression, we observed that the proportions of the B cells memory (*p* = 0.024), NK cells resting (*p* = 0.043), NK cells activated (*p* = 0.034), monocytes (*p* = 0.031), macrophages M2 (*p* = 0.002) and neutrophils (*p* = 0.015) significantly different between the TME of cluster1 and cluster2. We also observed that, compared to cluster2, in cluster1, the relative abundance of B cells memory, NK cells resting, macrophages M2 and neutrophils was lower, while the relative abundance of NK cells activated and monocytes was higher, emphasizing the importance of these infiltrated cells during the initiation and development of glioma. In general, Cluster1 which was associated with a good prognosis, was mainly infiltrated by immune‐activated cells like activated NK cells and monocytes, while Cluster2 which was associated with poor prognosis, was mainly infiltrated by immune‐inhibitory cells like macrophages M2. This result partly explained why Cluster1 had a strong survival advantage over Cluster2 as shown in Figure 2B.

In addition, we also scored the tumor microenvironment between these two clusters, including Immune Score, Stromal Score and ESTIMATE Score. The Stromal Score (Figure 5B; *p* = 4.1E‐12), ESTIMATE Score (Figure 5C; *p* = 8.8E‐12) and Immune Score (Figure 5D; *p* = 5.9E‐11) in Cluster2 were significantly higher than that in Cluster1, which indicated the higher infiltration of mesenchymal cells and immune cells, and thus the lower purity of tumor in Cluster2. Recently, relevant studies have proved that the survival prognosis is positively correlated with tumor purity.^54^ These results suggested that in cluster2, a high degree of immunosuppression‐related cell infiltration resulted in low tumor purity and poor survival prognosis.

### 3.5 The TMB analysis in distinct patterns of m6A‐DELPF expression

Defined as the total number of somatic gene coding errors, base substitutions, gene insertion or deletion errors detected per million bases, tumor mutation burden (TMB) is a kind of biomarker that can predict the patient's response to immunotherapy, because theoretically, the higher the TMB, the more antigens that can be recognized by T cells, and those who receive immune checkpoint inhibitor therapy may be with better prognosis.^55^ Herein, we analyzed the TMB between cluster1 and cluster2. We found that the TMB in cluster2 was significantly higher than that in cluster1 (Figure 5E, *p* = 1.2E‐11), suggesting that the efficacy of immunotherapy in cluster2 was better than that in cluster1. Specifically, in cluster1, the top five genes with mutation frequency were IDH1(59%),TP53(45%),ATRX(30%),CIC(15%) and PTEN(12%), while in cluster2, the top five genes with mutation frequency were TP53(25%),TTN(24%), PTEN(19%),EGFR(18%) and MUC16(13%) (Figure 5F, G). Additionally, the mutation of MUC16, SPTA1, TTN and RB1 in cluster1 were significantly lower than those in cluster2, while the mutation of ATRX, IDH1, CIC and TP53 were significantly higher than those in cluster2 (Figure S1A). Interestingly, most gene mutations are missense mutations in both cluster1 and cluster2, while only ATRX showed a higher proportion of the frameshift mutation in cluster1, especially the frameshift deletion (Figure S1A). There was no significant difference between cluster1 and cluster2 for the other genes, such as PIK3CA, FUBP1, NF1, PIK3R1, EGFR, PKHD1, PTEN, HMCN1, LRP2, PCLO, FLG and RYR2 (Figure S1A).

### 3.6 Construction of m6A‐DELPF signature (m6A‐DELPFS) and a corresponding nomogram to predict the prognosis of glioma

**FIGURE 6:**
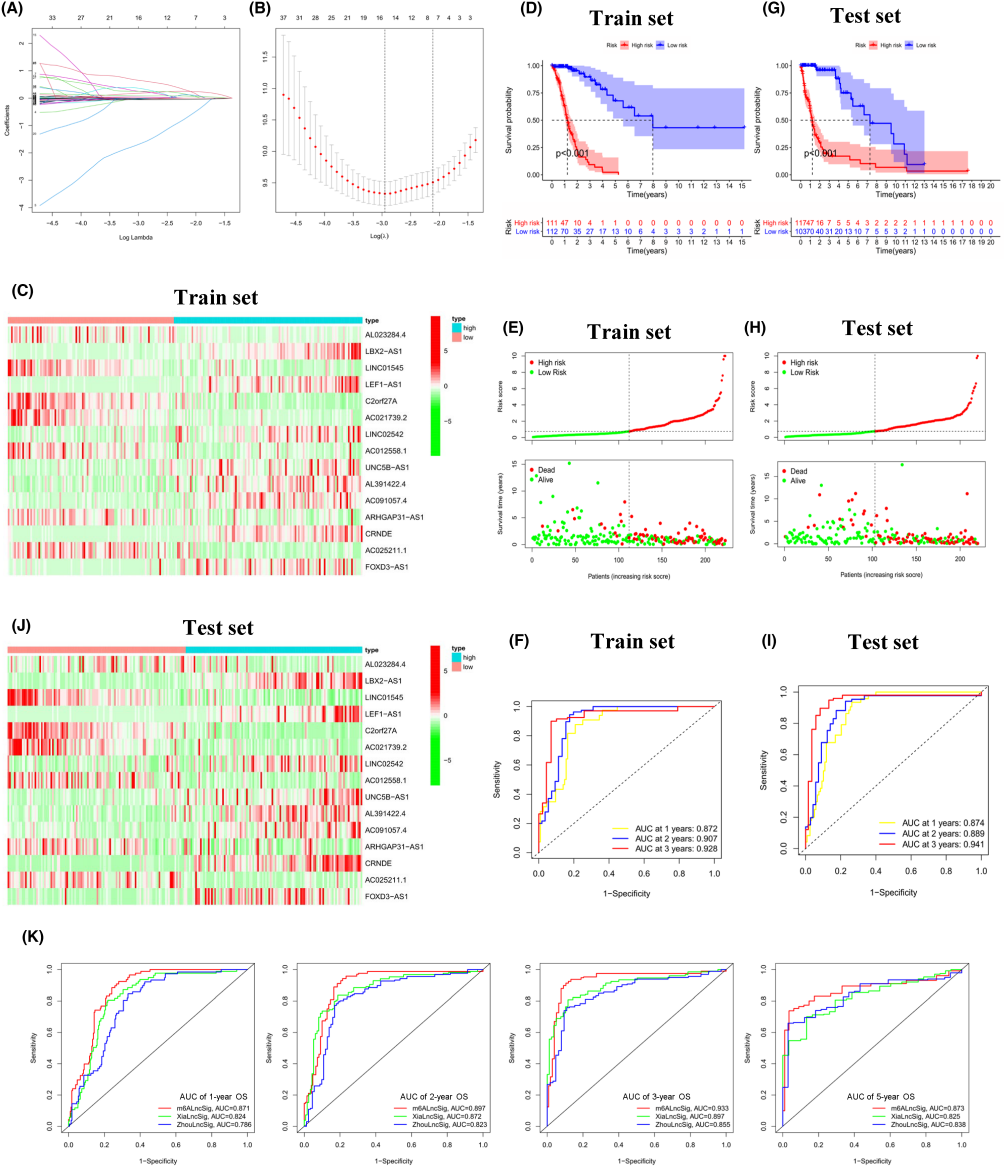
Construction of m6A‐DELPF signature(m6A‐DELPFS). (A,B) Least absolute shrinkage and selection operator (LASSO) regression was performed to calculate the minimum standard. (C) Heatmap of the expression of the selected 15 m6A‐DELPF in high‐risk group (bule) and low‐risk group (red) of training set. (D) Kaplan–Meier curves showed that the low‐risk group had better overall survival than the high‐risk group in training set of TCGA cohort. In the two‐sided log‐rank test, *p* < 0.05 is considered statistically significant. At the bottom of the chart, the grouping of the samples in train set is indicated. (E) Distributions of risk score and survival status of patients in training set. (F) ROC curves of m6A‐DELPFS for predicting the 1/2/3‐year survival in the train set. (G) Kaplan–Meier curves showed that the low‐risk group had better overall survival than the high‐risk group in testing set of TCGA cohort. In the two‐sided log‐rank test, *p* < 0.05 is considered statistically significant. (H) Distributions of risk score and survival status of patients in testing set. (I) ROC curves of m6A‐DELPFS for predicting the 1/2/3‐year survival in the testing set. (J) Heatmap of the expression of the selected 15 m6A‐DELPF in high‐risk group (bule) and low‐risk group (red) of testing set. (K) Comparing the ROC curves of m6A‐DELPFS and others' signatures from 1/2/3/5‐year overall survival was to confirm the stable and powerful prognostic ability of m6A‐DELPFS

According to the principle of random grouping, we divided the 443 samples in TCGA into the training set and testing set randomly, and the randomness of the grouping was confirmed using chi‐square test to see if gender, age, IDH gene mutation status and WHO grade were different between the two sets (Table S11). In order to construct m6A‐DELPFS and predict the OS of glioma patients, we performed Lasso Cox regression analysis in the TCGA cohort based on the 55 m6A‐DELPF previously obtained, and generated m6A‐DELPFS. This prognostic signature includes 15 m6A‐ DELPF and the respective coefficients (Figure 6A,B, Table S13). The expressions of these 15 lncRNAs in the high‐risk group and the low‐risk group are displayed in the heat map (Figure 6C).In the training set, the Kaplan–Meier survival curve showed that glioma patients with a lower risk score had longer overall survival, a better overall survival rate and better prognosis, compared with patients who had a higher risk score (Figure 6D; *p* < 0.001). In addition, the correlation between the specific risk score and survival status of patients with glioma also revealed that low‐risk patients did have a better prognosis than high‐risk patients (Figure 6E). The ROC curve in the training set also showed that this m6A‐DELPFS did have the potential capability to predict the OS of patients with glioma (Figure 6F, AUC at 1 year: 0.872, AUC at 2 year:0.907, AUC at 3 year:0.928). To further verify our conclusions in the training set, we performed the same analysis on the test set. Consistently, the results also showed that patients with higher risk scores had worse clinical prognostic outcomes (Figure 6G,H). We also analyzed the ability of this prognostic signature to predict OS in the testing set. The ROC curve verified the conclusions drawn in the training set (Figure 6I, AUC at 1 year: 0.874, AUC at 2 year: 0.888, AUC at 3 year: 0.941). The heat map showed the expression of 15 specific lncRNAs in the high and low‐risk groups in the testing set (Figure 6J). In recent years, there have been many studies using lncRNA to construct signatures that predict the OS of patients with glioma. For example, Pengfei xia et al. used 11 immune‐related lncRNAs to predict the survival of patients with glioma, and Peng Zhou et al. used 6 immune‐related lncRNAs to predict the prognostic characteristics of glioblastoma (Table S14). After comparing m6A‐DELPFS with their studies, we found that regardless of 1‐year OS, 2‐year OS, 3‐year OS or 5‐year OS in the ROC curve, m6A‐DELPFS had a more powerful and robust ability to predict the prognosis of patients with glioma (Figure 6K).

**FIGURE 7:**
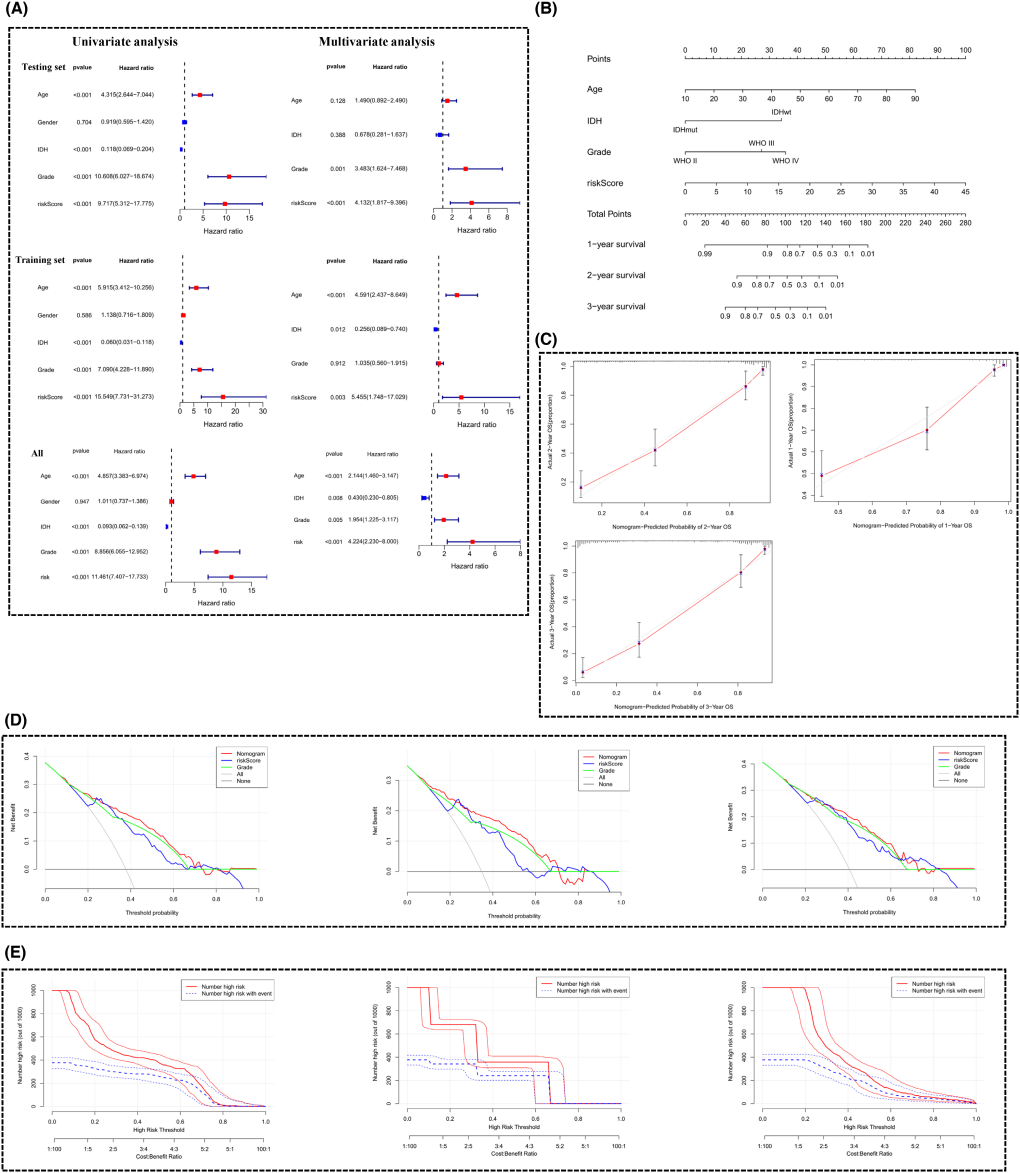
The m6A‐DELPFS is an independent, powerful and stable prognostic predictor. (A) Univariate and multivariate Cox regression model analysis, which included the factors of age, gender, IDH, grade and risk score in the test set, the train set and all. These revealed that risk score based on the m6A‐DELPFS was an independent prognostic predictor in the train and test sets. The 95% confidence interval (CI) is shown by the length of the horizontal line for each group. The vertical dotted line represents the hazard ratio (HR) of all patients shown in the forest plot. *p* < 0.05 is considered statistically significant. (B) Nomogram predicting the 1‐, 2‐ and 3‐year survival rates in patients with glioma. (C) Calibration plot of the nomogram for predicting 1‐, 2‐, and 3‐year OS for patients with glioma. (D) Decision curve analysis comparing the efficacy of nomogram, m6A‐DELPFS alone and traditional WHO grade in training, testing and overall sets. (E) Clinical impact curve evaluating the predictive performance and the value of the nomograms, m6A‐DELPFS alone and traditional WHO grade

Based on the data of glioma patients in the training set, we found that m6A‐DELPFS was a reliable and independent predictor of the prognosis (univariate: HR: 15.549, 95% CI: 7.731–31.273, *p* < 0.001; multivariate: HR: 5.455, 95% CI: 1.748–17.029, *p* = 0.003; Figure 7A). In the testing set, based on the combined results of univariate and multivariate regression analysis, we were able to rigorously demonstrate that m6A‐DELPFS was significantly associated with patient OS and could be used as an independent predictor of patient OS (univariate: HR: 9.717, 95% CI:5.312–17.775, *p* < 0.001; Multivariate: HR: 4.132, 95% CI: 1.817–9.396, *p* < 0.001; Figure 7A). Then we further verified the clinical independence in the overall sample containing the training set and the test set(univariate: HR: 11.461, 95% CI: 7.407–17.733, *p* < 0.001; Multivariate: HR: 4.224, 95% CI: 2.230–8.000, *p* < 0.001; Figure 7A). In general, these analyses indicated that m6A‐DELPFS could be utilized as an independent clinical prognostic signature. Moreover, we developed a prognostic nomogram to predict the overall survival rate for 1‐year, 2‐ year, and 3‐ year in the patients with glioma (Figure 7B) with the bias‐corrected C‐index of 0.835. The established calibration curve of 1‐year, 2‐year, 3‐year prediction confirmed the efficacy of the nomogram. (Figure 7C) Furthermore, a decision curve analysis (DCA) calculating the net benefit of using WHO grade, m6A‐DELPFS alone and the nomogram among training, testing and overall datasets were also performed (Figure 7D). Further clinical impact curves (CIC) obtained based on the overall datasets also showed the superiority of the constructed nomogram compared to m6A‐DELPFS alone and WHO grade (Figure 7E). Both of them revealed the excellent prognostic reliability and independence of the nomogram to predict the prognosis in patients with glioma, helping physicians analyze the clinical characteristics of each patient and use this as a reference for personalized medicine and clinical decisions.

### 3.7 The stratified analysis of m6A‐DELPFS based on clinicopathological characteristics

**FIGURE 8:**
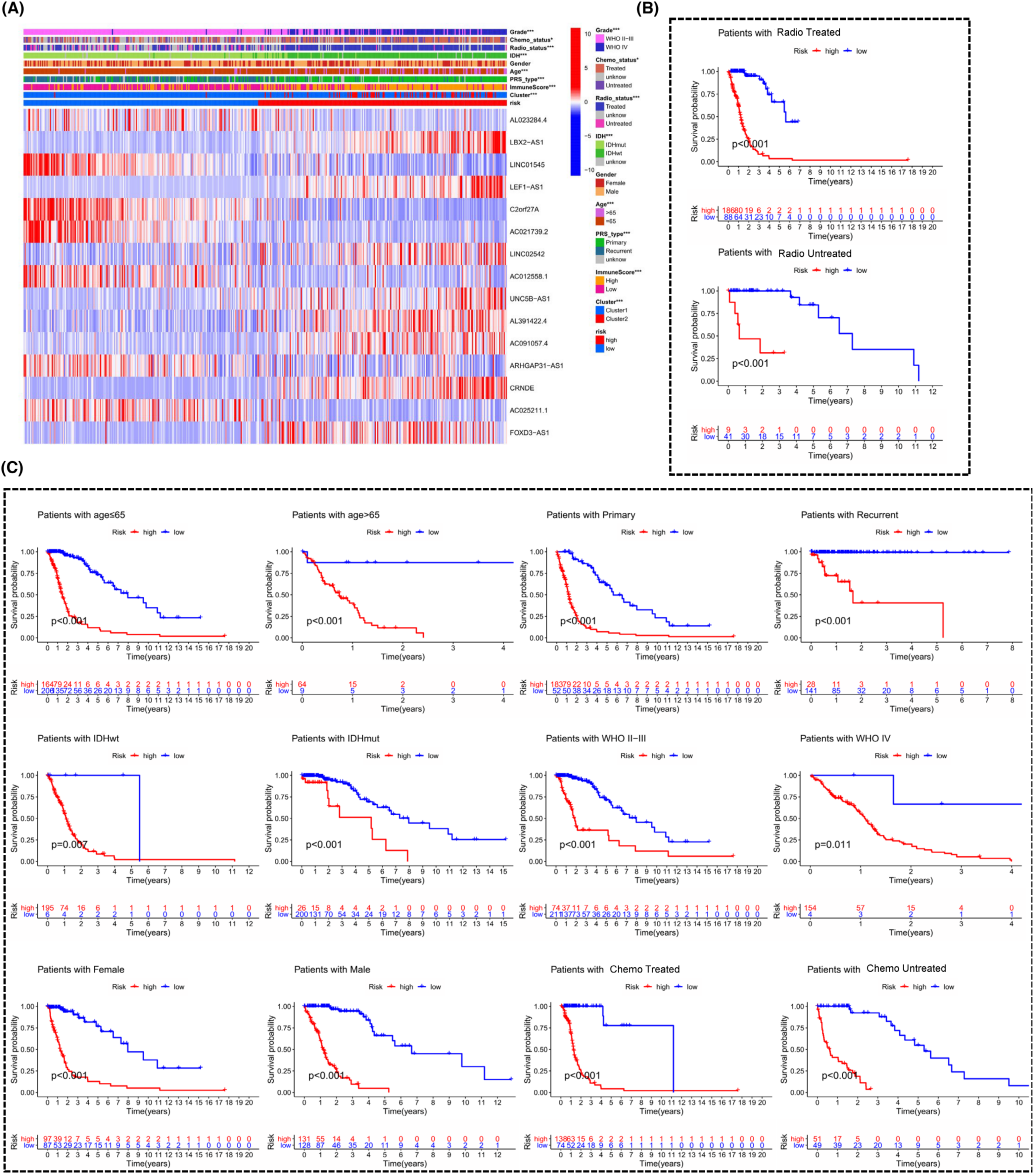
Stratified analyses showed the promising prognostic value of m6A‐DELPFS in different subgroups. (A) Heatmap visualizing the gene expression of the 15 m6A‐DELPF in high‐risk subgroup and low‐risk subgroup, and displaying associations between risk score based on the m6A‐DELPFS and multiple clinicopathological features. *p < 0.05, **p < 0.01 and ***p < 0.001. *p* < 0.05 is considered statistically significant. (B,C) The m6A‐ DELPFS retained its prognostic value in multiple subgroups of patients with glioma. These subgroups include patients aged ≤65 or > 65 years, patients with primary or recurrent glioma, patients with mutant or wild‐type IDH, patients with WHO grade II‐III or IV, patients with male and female, patients with or without chemotherapy treatment, and patients with or without radiotherapy treatment. *p < 0.05, **p < 0.01 and ***p < 0.001. *p* < 0.05 is considered statistically significant

We tried to confirm whether m6A‐DELPFS containing 15 lncRNAs was linked to clinicopathological features. The heat map presented that the risk score was positively relevant with the expression levels of LBX2‐AS1, LEF1‐AS1, LINC02542, UNC5B‐AS1, AL391422.4, AC091057.4, CRNDE and FOXD3‐AS1, while was relevant correlated with the expression of AL023284.4, LINC01545, C2orf27A, AC021739 0.2, AC012558.1, ARHGAP31‐AS1 and AC025211.1 (Figure 8A). The heat map also showed significant correlations between risk score and multiple clinicopathological features of glioma, such as WHO grade, chemotherapy status, radiotherapy status, IDH mutation status, age, PRS type, and immune score (Figure 8A). Specifically, patients with age >65 years old, radiotherapy treatment, chemotherapy treatment, WHO IV tumor, cluster2, high immune score, primary tumor, and wild‐type IDH had a higher risk score (Figure S2A–H), and risk score was not significantly correlated with gender (Figure S2I). Additionally, we performed a stratified analysis for each subset. Results showed that patients with a higher risk score had a shorter OS time and worse prognosis, both in the subset with age >65 years and with age ≤65 years (Figure 8B,C). Additionally, we also confirmed the strongly and steadily predictive ability of m6A‐DELPFS for glioma patients in multiple other subsets, including primary tumor subset, recurrent tumor subset, IDH wild‐type subset, IDH mutant subset, WHO II‐III subset, WHO IV subset, male subset, female subset, subset with chemotherapy treatment, subset without chemotherapy treatment, subset with radiotherapy treatment, and subset without radiotherapy treatment (Figure 8B,C). Conclusively, these data indicated that m6A‐DELPFS was indeed a stable and powerful prognosis predictor for patients with glioma.

### 3.8 Process and pathway enrichment via GO and KEGG based on the DEGs between high‐ and low‐risk group

**FIGURE 9:**
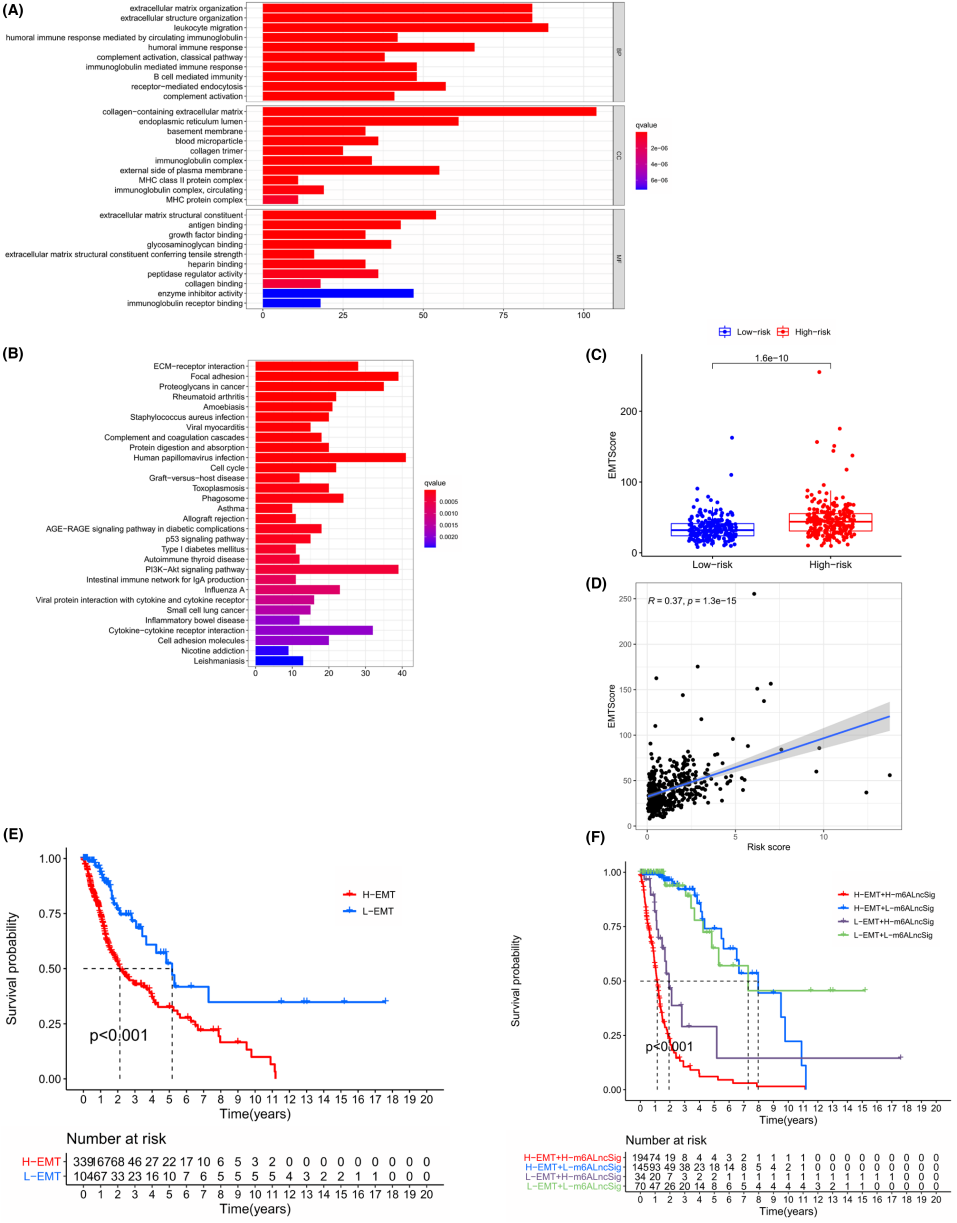
Functional enrichment analysis of 1455 differentially expressed genes(DEGs) and EMT analysis in high‐risk set and low‐risk set. (A) Colored according to q‐value, the bar plot was obtained via Gene Ontology(GO), visualizing the activation states of biological pathways molecular function and cellular component where the DEGs were enriched. (B) Obtained from KEGG and colored according to q‐value, the bar plot showed the signal pathways where these DEGs were enriched. (C) The patients with in different risk‐score sets had a significantly different level of EMT score. *p* < 0.05 is considered statistically significant. (D) The EMT score was positively correlated with risk score (*R* = 0.37, *p* = 1.3e‐15). (E) Kaplan–Meier curves showing overall survival of patients in high EMT‐score set or low EMT‐score set. In the two‐sided log‐rank test, *p* < 0.05 is considered statistically significant. At the bottom of the chart, the grouping of the samples is indicated. (F) Kaplan–Meier curves show overall survival of patients in high EMT‐score and high risk‐score set, high EMT‐score and low risk‐score set, low EMT‐score and high risk‐score set, or, low EMT‐score and low risk‐score set. In the two‐sided log‐rank test, *p* < 0.05 is considered statistically significant. At the bottom of the chart, the grouping of the samples is indicated

We screened and identified 1455 DEGs in the high‐risk and low‐risk sets (Table S15; |logFC| >1.5, FDR <0.001). Utilizing GO analysis, we found that those DEGs were mainly enriched in the following aspects: extracellular matrix organization, extracellular structure organization, leukocyte migration, humoral immune response and receptor‐mediated endocytosis, etc. (BP); collagen‐containing extracellular matrix, endoplasmic reticulum lumen, external side of plasma membrane, blood microparticle and immunoglobulin complex, etc. (CC); extracellular matrix structural constituent, antigen binding, enzyme inhibitor activity, glycosaminoglycan binding and peptidase regulator activity, etc. (MF) (Figure 9A; Table S16). Using KEGG analysis, we also found those DEGs were enriched in the following pathways: human papillomavirus infection, focal adhesion, PI3K‐AKT signaling pathway, proteoglycans in cancer and cytokine‐cytokine receptor interaction, etc. (Figure 9B; Table S17). Furthermore, those DEGs also were enriched in cell adhesion molecules, which played the core role in the epithelial‐mesenchymal transformation (EMT) of the tumor. Multiple researchers had demonstrated that the EMT was a key regulator associated with cancer invasion and metastasis.^56^, ^57^ On the whole, many of these DEGs were enriched in immune‐related signaling pathways or physiological processes, indicating that the immune microenvironment of tumors in the two different risk sets might be heterogeneous. The results of those DEGs enrichment analyses increased our understanding of biological processes at the molecular level such as the occurrence of glioma, immunosuppression, metabolic status, and tumor metastasis, etc.

### 3.9 Analysis of EMT score between the high‐ and low‐risk group based on m6A‐DELPFS

As mentioned above, EMT could promote the infiltration of glioma stem cells (GSC), thereby promoting the spread and infiltration of glioma. So we used 52 genes as mesenchymal markers and 25 genes as epithelial markers to analyze, and we finally got a summary table of EMT scores containing 703 samples (Table S19). We found that the EMT score in the high‐risk set was significantly higher than that in the low‐risk set (Figure 9C, *p* = 1.6e‐10). Moreover, we also found that the EMT score was positively relevant with the risk score \ (Figure 9D; *R* = 0.37, *p* = 1.3e‐15). We used the best cutoff to divide the samples into high EMT or low EMT set according to their EMT score. The survival analysis revealed that the patients with high EMT score had a worse prognosis with shorter overall survival time (Figure 9E, *p* < 0.001; Table S19). We attempted to combine the EMT score with m6A‐DELPFS risk to analyze the prognosis of the samples. The risk curve displayed that the glioma patients with high EMT scores and high m6A‐DELPFS risk had the shortest OS time (Figure 9E, *p* < 0.001; Table S19).

### 3.10 Analysis of the immune microenvironment of the glioma between the high‐ and low‐risk group based on m6A‐DELPFS

GO and KEGG showed the DEGs were mainly enriched the immune‐linked pathways. We compared the immune TME in the high‐risk set and the low‐risk set from multiple perspectives such as TMB score, immune infiltration and immunosuppressive gene expression.

**FIGURE 10:**
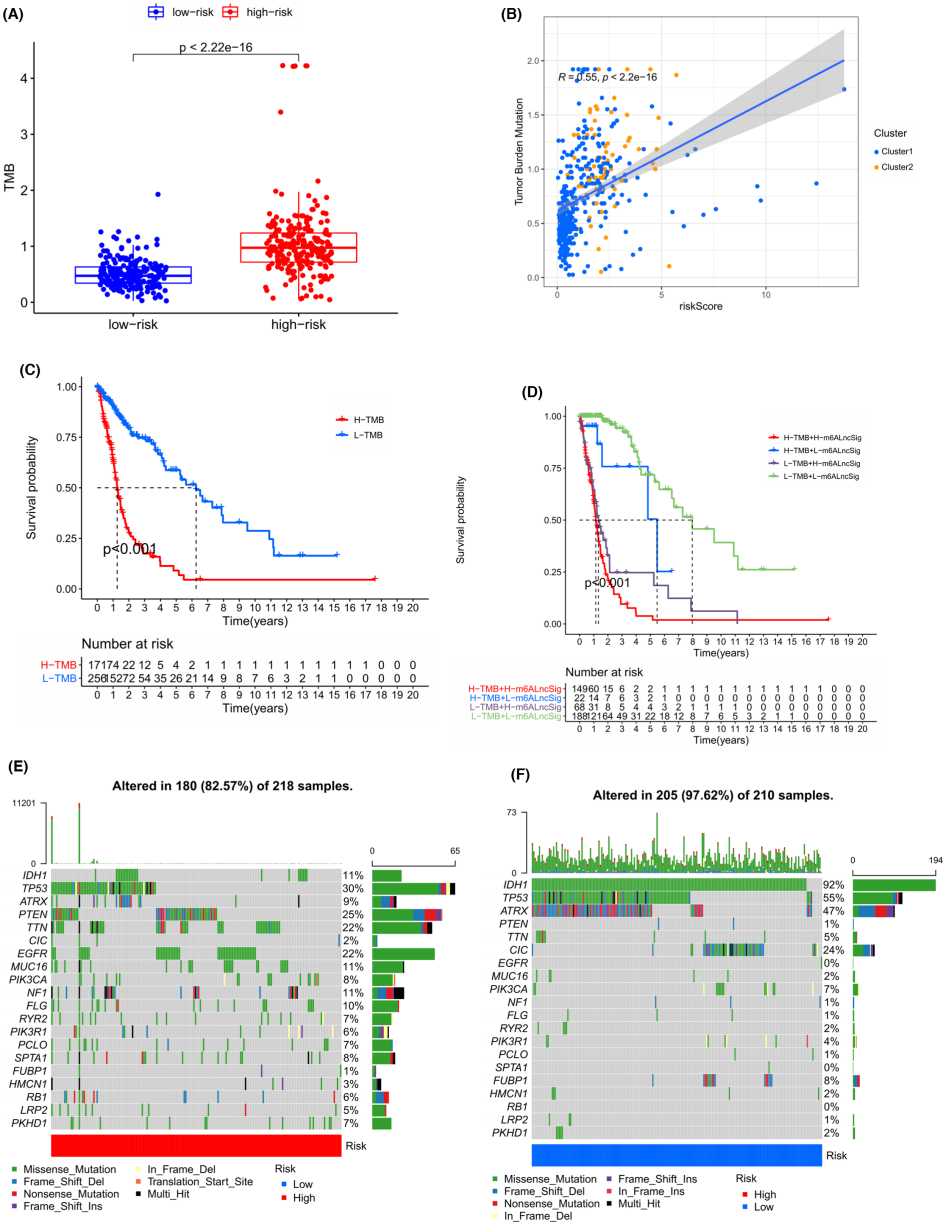
The genetic alteration of the samples in high‐risk set and low‐risk set. (A) Boxplot of different levels of TMB between the patients in high‐ and low‐ risk sets. *p* < 0.05 is considered statistically significant. (B) Correlation analysis between The TMB and risk score. In the Pearson correlation test, *p* < 0.05 is considered statistically significant. (C) Kaplan–Meier curves show overall survival of patients in high TMB set or low TMB set. In the two‐sided log‐rank test, *p* < 0.05 is considered statistically significant. (D) Kaplan–Meier curves show overall survival of patients in high TMB and high risk‐score set, high TMB and low risk‐score set, low TMB and high risk‐score set, or, low TMB and low risk‐score set. In the two‐sided log‐rank test, *p* < 0.05 is considered statistically significant. (E) The mutation frequency of the top 20 genes in 218 samples from high‐risk set of the TCGA cohort. Each column represents individual patients. The upper bar graph shows TMB; the number on the right indicates the mutation frequency in each gene. The right bar graph shows the proportion of each variant type. (F) The mutation frequency of 20 genes in 210 samples in low‐risk set from the TCGA cohort

As for the TMB score, we scored 443 samples in the previously processed cohort and got a TMB score summary table containing 428 samples due to missing data (Table S20). We found that the TMB score in the high‐risk set was significantly higher than that in the low‐risk set (Figure 10A, *p* < 2.22e‐16). Additionally, the positive correlation of TMB score and risk score was shown in Figure 10B (*R* = 0.55, *p* < 2.22e‐16). The survival curve in Figure 10C revealed that the patients with low TMB scores had better prognosis (*p* < 0.001). The survival curve in Figure 10D showed that the patients with low TMB score and low risk score had the longest OS time and the best prognosis (*p* < 0.001). Moreover, we summarized the genes of the high‐risk set and low‐risk set whose mutation contributed to TMB score respectively in Figure 10E,F. According to mutation frequency, the top four genes are TP53(30%), PTEN (25%), TTN (22%) and EGFR (22%) in high‐risk set and IDH1 (92%), TP53 (55%), ATRX (47%) and CIC (24%) in low‐risk set. We also found that the missense mutation accounted for the majority of mutations in almost every gene except ATRX. In terms of specific genes mutation in the high‐risk set and the low‐risk set, there was no significant difference in mutation of LRP2, PIK3CA, HMCN1 and PIK3R1 (Figure S3A). However, the mutation percent of RB1, PTEN, PKHD1, PCLO, NF1 and MUC16 in the high‐risk set was significantly higher than that in the low‐risk set, while the mutation percent of IDH1 and FUBP1 was opposite (Figure S3B).

**FIGURE 11:**
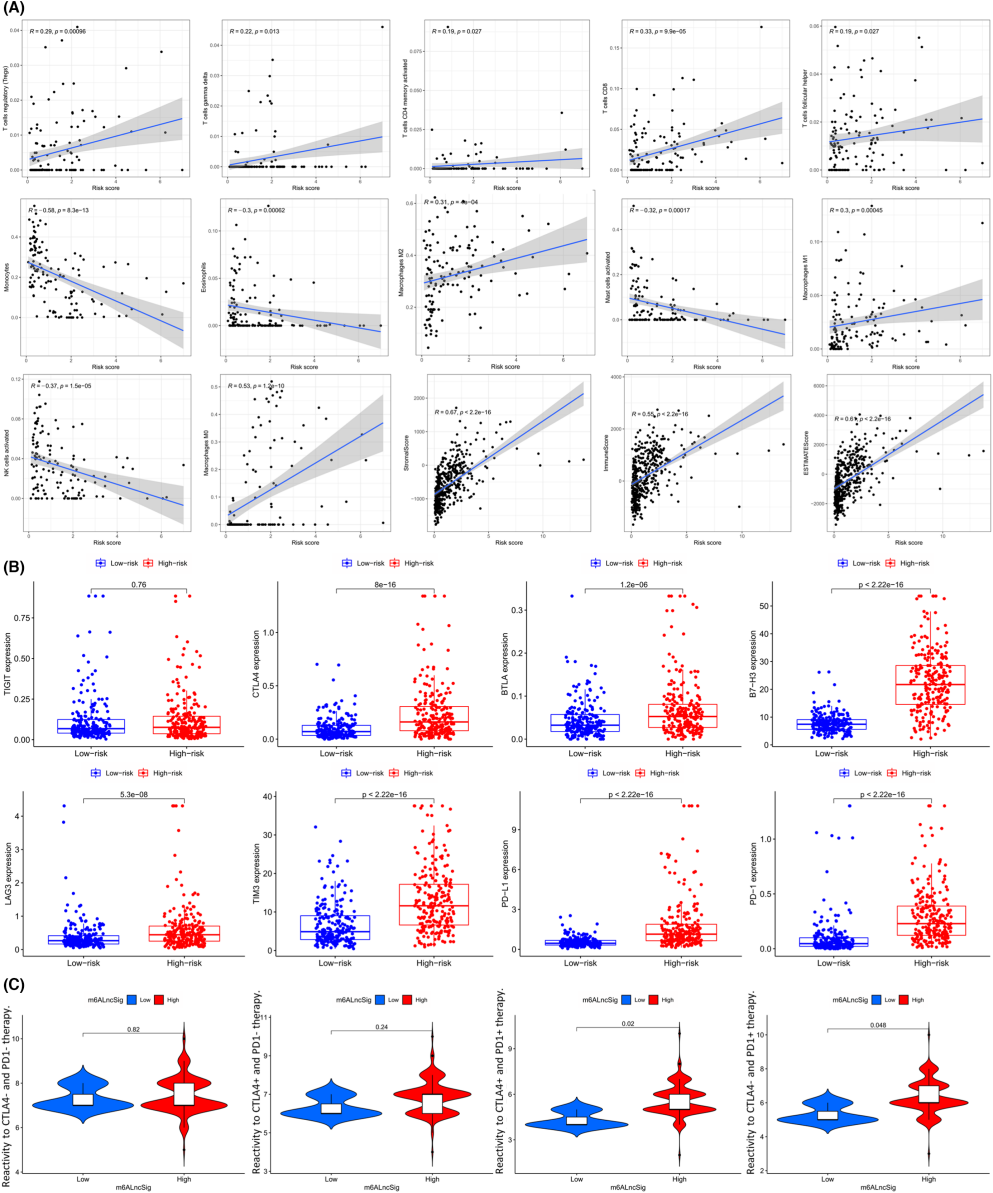
Analysis of the immune microenvironment of the glioma and immunotherapy sensitivity. (A) Infiltration of Tregs, T cells gamma delta, T cells CD4 memory activated, T cells CD8, T cells follicular helper, macrophages M2, macrophages M1, macrophages M0, the stromal score, immune score and ESTIMATES score are positive related with the risk score, while infiltration of monocytes, eosinophils, mast cells activated and NK cells activate is negatively associated with the risk score. *p* < 0.05 is considered statistically significant. (B) The boxplots display differently expressed immune checkpoint genes in patients with high‐risk set (red) and low‐risk set (blue). *p* < 0.05 is considered statistically significant. (C) The violin plots showing different level of drug response to CTAL‐4 and PD‐1 blocking immunotherapy in patients with high‐ or low‐ risk. *p* < 0.05 is considered statistically significant

In the tumor microenvironment, it seems that a variety of immune‐related cells are the most important, such as macrophages M1, CD8+ T cells, activated NK cells, etc. These cells secrete various factors that affect the microenvironment outside the tumor and regulate tumor behavior. Herein, as for immune infiltration, we accessed evaluated the relationship between some immune cells and risk scores. The results indicated that the risk score was positively related with the infiltration of Tregs, gamma delta T cells, activated CD4 memory T cells, CD8 T cells, T cells follicular helper, and macrophages M2, while was negatively associated with the infiltration of monocytes, eosinophils, mast cells activated, and NK cells activate (Figure 11A). Besides, we also found that the risk score was positively relevant with the stromal score, immune score and ESTIMATES score (Figure 11A).

And then, we compared the difference of the immunosuppressive genes in the sets and found that the gene expression of CTLA4, BTLA, B7‐H3, LAG3, TIM3, PD‐L1 and PD‐1 in the high‐risk set was significantly higher than that of the corresponding gene in the low‐risk set (Figure 11B). Besides, TIGIT (*p* = 0.76) seemed to have no significant relationship with risk score.

### 3.11 The m6A‐DELPFS can predict the efficacy of the anti‐PD‐1 and anti‐CTLA4 immunotherapy and aid in the identification of putative chemotherapeutic drugs

**FIGURE 12:**
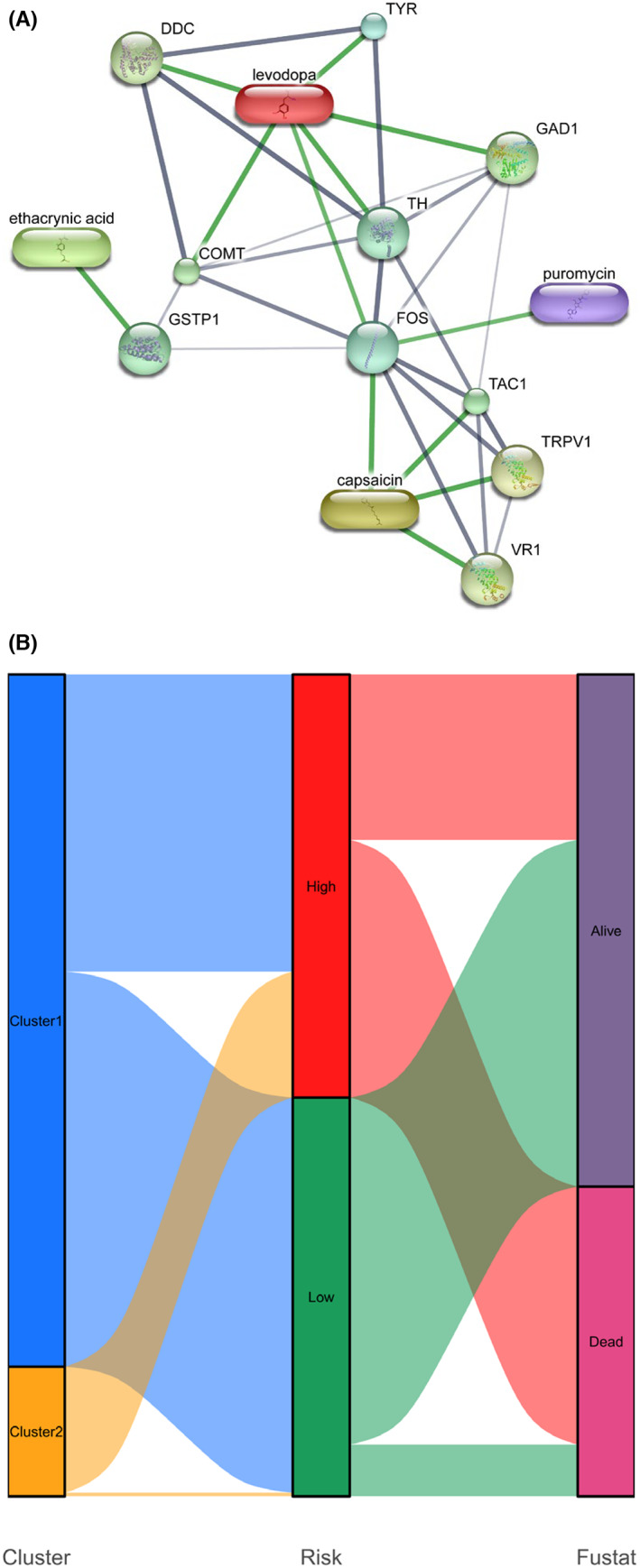
The molecules targeted by the predicted drug and the relationship of cluster 1/2, high/low‐risk set and survival outcomes. (A) The network of the predicted drugs and the potential targeted molecules. The width of edges represents the confidence score. The thicker the line, the stronger the correlation between the two entities. Protein–protein interactions are depicted in gray. Chemical‐protein interactions are depicted in green. (B) Sankey diagram of the relationship of the cluster 1/2, high/low‐risk set and survival outcomes (alive/dead)

We compared the TMB of the two sets before, and the patients in the high‐risk set had the higher TMB, indicating the good immunotherapy effect. Here we used the TCIA database to predict the reactivity of the patients in two sets to the immunotherapy targeting CTLA‐4 and PD‐1(Table S21). The results revealed that the responses of the high‐risk set to the immunotherapy were significantly higher than the low‐risk set when targeted drugs for PD‐1 and targeted drugs for CTLA‐4 were used in combination (Figure 11C; *p* = 0.02) or when targeted drugs for PD‐1 was used and targeted drugs for CTLA‐4 was not utilized (Figure 11C; *p* = 0.048). Interestingly, when both targeted drugs for PD‐1 and targeted drugs for CTLA‐4 were not used (Figure 11C; *p* = 0.82) or when targeted drugs for PD‐1 was not used and targeted drugs for CTLA‐4 was utilized (Figure 11C; *p* = 0.24), the difference of reactivity of the patients in two set was not significant. This means that low‐risk and high‐risk patients had the same sensitivity to anti‐CTLA4 monotherapy. Moreover, high‐risk patients were more responsive to anti‐PD‐1/CTLA4 combination therapy and anti‐PD‐1 monotherapy. Therefore, segmentation in the population with glioma will help these groups to find more suitable sub‐therapies. In addition to CTLA‐4 and PD‐1, we utilized the cMAP datasets to explore the potential therapeutic drugs targeting the high‐risk glioma, and we got 20 prospective drugs with a significant score for glioma treatment including 5,182,598, exisulind, MG‐262, etc. (Table S22; enrichment <−0.5, *p* < 0.05). To determine the common proteins directly targeted by these drugs, we also used STITCH database to predict the upstream proteins directly targeted by these drugs (Figure 12A). The GO and KEGG analysis of the downstream proteins showed that those prospective drugs for glioma treatment possibly took effect via affecting pathways including tyrosine metabolism, dopaminergic synapse and so on (Table S23). Collectively, the above results showed that the efficacy of both immunotherapy and chemotherapy can be predicted based on the m6A‐DELPFS.

All in all, the patient groups divided by the k‐means clustering or m6A‐DELPFS showed a bunch of similar characteristics. The Sankey diagram was shown in Figure 12B to illustrate the correlation among different groups. Results showed that patients in cluster1 can also be further divided into high and low‐risk group, the reason of which was that the risk score of each patient calculated by m6A‐DELPFS may still vary internally because m6A‐DELPFS was only based on 15 genes while the unsupervised clustering was based on up to 55 genes. The further division confirmed the heterogeneity of patients in cluster1.

### 3.12 Construction of the ceRNA Network and Functional Enrichment Analysis

**FIGURE 13:**
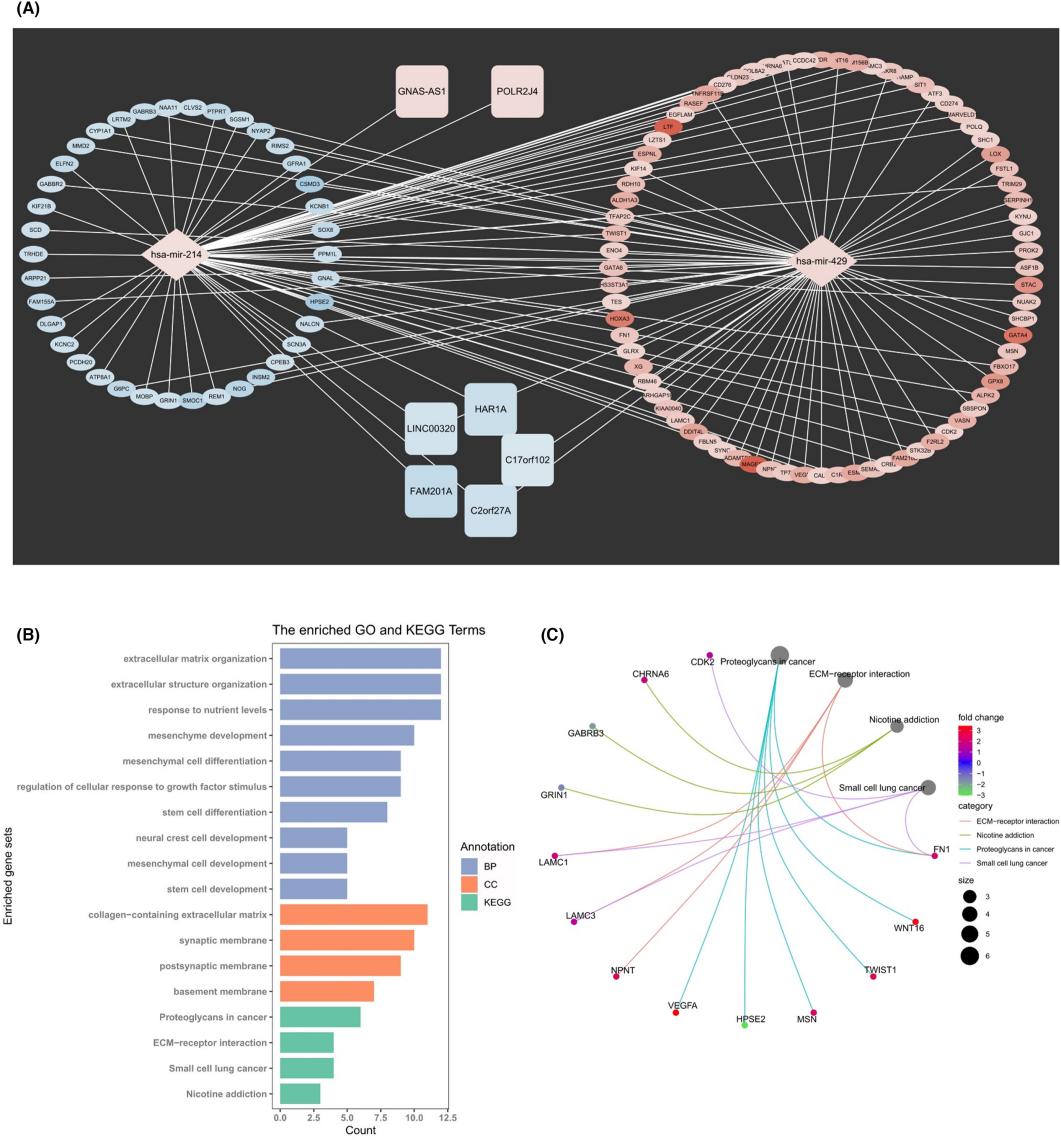
Construction of ceRNA network based on different risk groups clustered by m6A‐DELPFS. (A) The ceRNA network of the seven m6A‐related lncRNAs and their target miRNAs and mRNAs. GNAS‐AS and POLR2J4 are upregulated lncRNAs (Red), while FAM201A, C2orf27A, LINC00320, HAR1A and C17orf102 are down‐regulated lncRNAs (Blue). The hsa‐mir‐214 and hsa‐mir‐429 are up‐regulated miRNA. The blue means downregulated and the red means up‐regulated. The oval shape represents mRNA, the square shape represents lncRNA, and the diamond shape represents miRNA. (B) The enriched GO and KEGG terms of target proteins in ceRNA network with adjusted *p* < 0.05. (C) The corresponding gene of the corresponding gene set and its fold change in the high‐risk group. The most likely oncogenes and the specific action pathways of lncRNA‐miRNA‐mRNA were obtained

The ceRNA network consisted of seven lncRNAs, two miRNAs and 114 mRNAs (Figure 13A). Through functional enrichment of downstream mRNA in the ceRNA network, the corresponding gene set is obtained (Figure 13B). We found those mRNAs were enriched in extracellular matrix organization, extracellular structure organization, response to nutrient level, mesenchyme development, mesenchymal cell differentiation, regulation of cellular response to growth factor stimulus, stem cell differentiation, neural crest cell development, mesenchymal cell development and stem cell development (GO Biological Processes); collagen‐containing extracellular matrix, synaptic membrane, postsynaptic membrane and basement membrane (GO cellular component); proteoglycans in cancer, small cell lung cancer and nicotine addiction and ECM‐receptor interaction(KEGG pathways). Through enrichment analysis, the genes corresponding to the gene set and their multiples of change in the high‐risk group were obtained (Figure 13C). According to the genes obtained by the enrichment analysis, the most likely oncogene was predicted, and the specific action pathway of lncRNA‐miRNA‐mRNA was obtained. Noticeably, the constructed predictive pathway can partially explain why high‐risk components are high‐risk. For instance, one of the enriched gene sets was ECM‐receptor interaction, which might be related to infiltration, EMT, etc. However, some events that we had found to be significantly related to survival, for example, TMB, immune infiltration, TME, etc., cannot be well explained by the predicted pathways. Interestingly, the pathway analysis in cluster 1 and cluster 2, and high‐ and low‐risk group showed the enrichment of immune‐related pathways (Figure 3 and Figure 9). However, functional enrichment of downstream mRNA in the ceRNA network did not confirm their correlation with those pathways. This phenomenon occurred may result from the fact that the interactions between lncRNA‐miRNA and miRNA‐mRNA were predicted using the intersectional data among miRcode, miRDB, miRTarBase, TargetScan database, some of which (e.g., miRTarBase) were based on the experimentally validated interaction information. Thus, the incomplete prediction may cause the inconformity of the functional enrichment of downstream mRNA in the ceRNA network. These results indicated that most regulatory mechanisms of this special lncRNA subgroup‐ m6A related lncRNAs‐ are still needed to be explored.

### 3.13 Validation analyses in external CGGA and GEO datasets confirmed the roles of m6A‐related lncRNAs in glioma

To screen the m6A‐related lncRNAs in CGGA cohort, similar processes in TCGA were conducted. The first step was to confirm the expression mode of 23 m6A related genes. The intersection between CGGA derived m6A‐related LncRNAs and TCGA derived m6A‐related LncRNAs showed a highly varied constitution in different cohorts (Figure S4A). An unsupervised clustering analysis (k‐means clustering) was performed in CGGA cohort with 500 repetitions to guarantee the stability of classification. The analysis also clustered the patients from CGGA into cluster1 and cluster2 (Figure S4B), indicating that the expression mode of m6A‐related lncRNAs is stable across different datasets. Cluster 1 showed a better prognosis than cluster 2 in patients with glioma (Figure S4C). We also analyzed the data from CGGA in the same way as the data from TCGA, including immune gene expressed analysis, cibersort analysis and so on. The expression of BTLA (*p* = 7.5e−05), PD‐L1 (*p* = 3.7e−05), B7 − H3 (*p* < 2.22e−16), TIM3 (*p* = 0.0037), CTLA4 (*p* = 0.00014), LAG3 (*p* = 0.00014) and PD − 1 (*p* = 7.4e−08) in cluster 1 and cluster 2 was significantly different (Figure S4D). In addition, the ESTIMATE Score, Immune Score, Stromal Score and EMT score in cluster 1 were significantly lower than those in cluster 2 in CGGA (Figure S5B–E), which was consistent with the results in TCGA. Those results partly indicated the stability of this classification based on m6A related lncRNAs. To confirm the efficacy of prognostic prediction value of m6A‐related lncRNAs clustering, we also performed the survival analysis in different clinical subgroups (Figure S6A–F). The results were highly consistent with those obtained in TCGA. It also showed that the m6A‐related lncRNAs had the stable ability to subdivide the prognosis of patients in various clinical contexts. In addition, utilizing GO analysis, we found that differentially expressed genes between cluster1 and cluster2 in CGGA were mainly enriched in the following gene sets: extracellular matrix organization, extracellular structure organization, etc. (BP); collagen‐containing extracellular matrix, etc. (CC); receptor ligand activity, signaling receptor activator activity, etc. (MF) (Figure S7A). Using KEGG analysis, we also found those DEGs were enriched in the following pathways: cytokine‐cytokine receptor interaction, PI3K‐AKT signaling pathway, etc (Figure S7B). The results of the GO and KEGG in CGGA were highly similar to those in TCGA (Figure 9A,B). Consistent with CGGA and TCGA, results in GSE108474 and GSE16011 showed similar clustering results (Figure S8,S9). The survival rates in Cluster 1 and Cluster 2 are also significantly different not only in the whole datasets, but also in different clinical stratification. Collectively, despite different constitutions of m6A‐related lncRNAs, follow‐up analysis with CGGA showed a highly consistent pathway with TCGA, indicating that: 1) The detection of m6A‐related lncRNAs in different sequencing platforms had certain differences.2) The m6A‐related lncRNAs played important roles in glioma, so their correlations with various pathways were highly consistent in different datasets.

## 4 DISCUSSION

Recent evidence indicated that m6A or lncRNAs is critical in the occurrence, proliferation, growth, metastasis, invasion, anti‐tumor immunity and poor prognosis of glioma. However, there are few studies on the role of m6A‐related lncRNAs in gliomas. Here, we identified 55 m6A‐related and differently expressed lncRNA with prognostic function, and then we got two sets cluster1 and cluster2 via k‐means clustering analysis based on the 55 m6A‐DELPF and constructed a prognostic signature including 15 m6A‐DELPF and each coefficient. The cluster1 was associated with a good prognosis, decreased expression of immunosuppressive checkpoints, increased infiltration of main immune‐activated cells and low TMB scores. We scored each sample based on the m6A‐DELPF signature to assess survival in each patient. The high‐risk set was linked to worse prognosis, increased expression of immunosuppressive checkpoints, increased infiltration of main immune‐suppressed cells, high TMB scores and high EMT score. No matter it was in cluster1 and cluster2, or in the high‐risk set and the low‐risk set, the abundance of immune infiltrating cells in the tumor microenvironment is significantly different.

The enrichment analysis via GO and KEGG revealed that the DEGs in the high‐risk set and low‐risk set were mainly enriched the immune‐linked pathways like leukocyte migration. The immune microenvironment of glioma, including infiltrating and resident immune cells, vascular cells and other glial cells, plays an extremely critical role in pathological processes such as tumor genesis, immunosuppression, immune escape and tumor metastasis, etc. Therapies targeting the immune microenvironment, such as immune checkpoint therapy and active immunization, have made remarkable achievements, suggesting that immune‐related molecules in the tumor microenvironment have the potential to be biomarkers for predicting the prognosis of patients with glioma.^58^, ^59^ Our results revealed the potential molecules and pathways of the immune microenvironment and the metabolic microenvironment. The patients with high risk were be considered with highly expressed immunosuppressive genes such as PD‐L1, PD‐1 and CTLA‐4. We tested the drug response of gliomas to PD‐1 and CTLA‐4, and the results showed that high‐risk gliomas are highly responsive to PD‐1 immunotherapy, but poorly to CTLA‐4 immunotherapy. In fact, in a series of available tumor immunotherapies, the PD‐1 monoclonal antibody blockade shows promising therapeutic potential.^60^, ^61^, ^62^, ^63^ A new randomized, multi‐institution clinical trial evaluating the immune response and survival in 35 patients with recurrent glioblastoma shows that neoadjuvant anti‐PD‐1 immunotherapy promotes both intra‐tumoral and systemic immune responses in patients with recurrent glioblastoma, resulting in improved survival.^64^ However, as predicted by our results, CTLA4‐based drugs for the treatment of gliomas have so far been rare due to the low drug responsiveness of gliomas to CTLA4. Our results indicated the potential of m6A‐DELPF to predict the sensitivity of patients to different immunotherapy regimens. The infiltration of the NK cells activate was negatively related with the risk score in our analysis. In fact, the NK cells can modulate the growth of the glioma via sensing growth factors and secreting interferon gamma and tumor necrosis factor alpha.^65^ In addition, studies have also proved that some specific NK cells can also be utilized in the targeted therapy of glioma.^66^ As the classical immunosuppressive cells, the infiltration of Tregs was positively associated with the risk score. The migration of Tregs and increased immune infiltration in the tumor microenvironment of glioma have been observed in several studies, and the immune infiltration of Tregs promotes growth, proliferation, metastasis and invasion of the glioma.^67^, ^68^ In addition to Tregs, we also found that the immunosuppressive cells, for example, macrophage M2, were also positively correlated with risk scores. However, we also observed that the infiltration of some immune‐activated cells such as macrophages M1, T cells CD8, T cells CD4 memory activated, were abnormally and positively correlated with risk score. The possible reason for this situation is that: 1) the sample size is not enough. 2) even these immune‐activated cells were enriched in the TME of high‐risk glioma, some underlying mechanisms hinder them from functioning properly. 3) The CIBERSORT algorithm is theoretical and not experimental. The expression of genes can also be overexpressed in cancer cells. The specific mechanisms of the disability of immune‐activated cells in TME still need to be further studied. Moreover, we performed the analysis of the TMB score and the results indicated that the high‐risk samples had a high TMB score and worse prognosis. Other studies on TMB have also confirmed that TMB is closely related to the poor prognosis of multiple types of glioma.^69^, ^70^ TMB is a biomarker for immunotherapy and prognosis, but for glioma the TMB is controversial. A recent study showed that high TMB does not predict immune checkpoint blockade in all types of glioma. Its results showed that in glioma, TMB‐high tumors failed to achieve 20% ORR (ORR = 15.3%), which is thought to cause poor immunotherapy effects.^71^ In general, the predictive effect of TMB on tumor immunotherapy is still full of controversy, which needs further research in the future. We summarized the gene mutation frequency in TMB. The top four genes are TP53 (30%), PTEN (25%), TTN (22%) and EGFR (22%) in high‐risk set and IDH1 (92%), TP53 (55%), ATRX (47%) and CIC (24%) in low‐risk set. In general, gliomas with IDH1 mutations have a good prognosis, but low‐grade gliomas with IDH mutations usually deteriorate to advanced gliomas, leading to a poor prognosis.^72^ Mutations in TP53 and PTEN can regulate the renewal and differentiation of glioma stem cells by driving the expression level of myc protein, promoting tumor progression and leading to poor prognosis.^73^, ^74^ ATRX, a chromatin remodeling protein, is considered to be associated with the prognosis of patients with glioma and a novel therapy target.^75^

In recent years, researchers have constructed signatures based on immune‐related genes to effectively and independently predict the overall survival of patients with glioma.^76^, ^77^, ^78^ We also analyzed the power of our signature compared with similar research, which further proved that m6A‐related lncRNA could indeed predict the prognosis of glioma in a more effective manner. We also confirmed the clinical independence of m6A‐DELPFS compared with other clinical parameters like WHO grade, age, IDH mutation status, etc. Based on the multivariate Cox regression analysis, we combined the m6A‐DELPFS and clinical parameters most relevant to survival to construct a clinically accessible nomogram. The subsequent calibration curve analysis, C‐index analysis, DCA analysis and CIC analysis all confirmed the superiority of nomogram in predicting prognosis compared to using WHO grade or m6A‐DELPFS alone.

We also uncovered the potential role of m6A‐DELPFS for therapy in glioma. Based on the m6A‐DELPFS, we explored glioma potential therapeutic drugs and got 20 perspective drugs. Then, we performed enrichment analysis on these drug‐targeting molecules and found they were enriched in tyrosine metabolism, dopaminergic synapse and amphetamine addiction, etc. Dysregulation of receptor tyrosine kinases plays a vital role in the progression of glioma.^79^, ^80^ After activation of EGFR, phosphorylation of tyrosine activates 6‐phosphoglucate dehydrogenase, with increased adverse prognosis and malignancy.^81^ As mentioned above, for the patients, the response to PD‐L1 blockade immunotherapy can be predicted by the risk score based on m6A‐DELPFS. Despite the lack of external data sets for verification, our results provide novel and promising possibilities for improving the treatment effect and prognosis of gliomas, mainly because different patients can be classified, identified, and then personalized precision medical treatment (surgical resection, immunotherapy, chemotherapy/ Radiotherapy, etc.), which not only guarantees the curative effect, but also relieves the economic pressure of patients to a certain extent.

Through the ceRNA network, we could find that the m6A‐related lncRNAs had a very high possibility to regulate downstream oncogenes through the two miRNAs (hsa‐mir‐214 and hsa‐mir‐429). Downstream target proteins including LAMC1, LAMC3, CDK2 and FN1 were enriched in the cancer‐related pathways in small cell lung cancer. Remarkably, the m6A‐related lncRNAs could regulate the expression of the four mRNA via the miRNAs. So our results revealed the specific pathways of the mechanism of how m6A‐related lncRNAs regulated the mRNA, providing the potential effective targets for drugs and clinical therapy in patients with glioma. Besides, judging from the number of lncRNA, there are still many m6A‐related lncRNA functions that have not been fully deciphered. At the very beginning, we identified1119 m6A‐related lncRNAs, 349 m6A‐related lncRNAs with prognostic function, 92 lncRNAs with significant differential expression between glioma and normal tissues and 55 differentially expressed m6A‐LPF. However, only 7 were found in the integrated database analyses to be effective in ceRNA network construction, which only included one lncRNA that is one of the m6A‐DELPFS, indicating that most regulatory mechanisms of this special lncRNA subgroup‐ m6A related lncRNAs‐ are still needed to be explored.

It's also worth noting that several limitations still existed in our present investigation. First, the m6A‐related lncRNAs were defined solely based on the expression mode of 23 pre‐identified m6A methylation genes including 8 m6A writers, 13 readers and 2 erasers. Due to the lack of m6A sequencing data in TCGA, the exact methylation status and methylation site of m6A‐related lncRNAs remained unknown. Second, the normal sample size in TCGA cohort was small (*n* = 5), which is one of the main defects of the data. To minimize the false‐positive rate from integrating multiple datasets, we choose to not introduce the normal samples from outer datasets but to directly perform the differential expression analyses utilizing the small‐size normal sample. Despite that, the statistical power of our analyses was still strong. Second, the m6A‐related lncRNAs varied between different datasets, making it hard for us to directly confirm the efficacy of m6A‐DELPFS in other datasets. To address this problem, we conducted the same unsupervised clustering method k‐means on the patients in CGGA to mimic m6A‐DELPFS based subdivision of patients. The results in CGGA confirmed the high correlation between m6A‐related lncRNAs and aforementioned pathways including EMT, tumor microenvironment alteration and immune infiltration. The clinical efficacy of the unsupervised clustering was also confirmed using clinical characterization analysis in CGGA, GSE108474 and GSE16011. Third, the analysis of reactivity to immunotherapy was based on TCIA databases and only TCGA data was utilized. As for other cohorts related to immunotherapy, the analysis was unable to conduct because of lacking the corresponding lncRNAs in other real immunotherapy responsiveness data. Despite the above limitations, the inclusion of these novel lncRNAs in the prognostic signature was still meaningful because it reflected that several m6A‐related lncRNAs existed with a high correlation with the prognosis and pathophysiology of glioma, but without further investigation. Our current investigation identified these novel lncRNAs and provided insights into their functions and pathways they may participate in the occurrence and progression of glioma.

## 5 CONCLUSION

Herein, our present research constructed a prognostic signature namely m6A‐DELPFS, consisting of the 15 m6A‐DELPF and respective coefficients. This prognostic model was evaluated and proved to show relatively high efficacy and independence in the prediction of glioma prognosis. Additionally, we predicted potential drugs and their possible molecular targets based on the model. Moreover, we proved that different clusters of m6A‐DELPF closely correlate with clinicopathological characteristics, immune checkpoints, tumor mutation burden and immune infiltration, which all contributed to the initiation and development of glioma. We also constructed a ceRNA network to look into the possible mechanisms downstream of m6A related lncRNAs in tumorgenesis. Collectively, our research provided insights into the clinical value and potential downstream mechanisms of m6A related lncRNAs in glioma. The actual clinical performance of m6A‐DELPFS and the corresponding nomogram, the possible mechanisms of m6A‐DELPF and the curative effect of the drugs in glioma still warranted further investigation.

## AUTHOR CONTRIBUTIONS

Conceptualization and formal analysis, C.Z.; writing‐original draft preparation, Z.W. and C.Z.; writing‐review and editing, Y.Z. and Z.M; supervision, funding acquisition and validation, Z.M.

## FUNDING INFORMATION

This research was funded by Research Project on Education and Teaching Innovation of Central South University (Granted number: 2021jy145), the Natural Science Foundations for Excellent Young Scholars of Hunan Province (Granted number: 2021JJ20095), the Natural Science Foundations of Hunan Province (Granted number: 2020JJ4134), the Key Research and Development Program of Hunan Province (Granted number: 2020SK2063), and the National Natural Science Foundation of China (Granted number: 81501025).

## CONFLICT OF INTEREST

The authors have declared that no competing interest exists.

## ETHICS APPROVAL AND CONSENT TO PARTICIPATE

Not applicable.

## CONSENT FOR PUBLICATION

Not applicable.

## Supporting information


Figure S1

Table S1

## Data Availability

Publicly available datasets were analyzed in this study. These data are available in the TCGA repository, https://portal.gdc.cancer.gov/; CGGA repository, http://www.cgga.org.cn/; TCIA repository, https://tcia.at/home.
