# Supplementary material for: Comprehensive analyses indicated the association between m6A related long non‐coding RNAs and various pathways in glioma
Source: Cancer Med. 2022 Jun 6;12(1):760–88. doi: 10.1002/cam4.4913 (PMC9844638; doi:10.1002/cam4.4913)
Supplement: Supplementary file 1 — Figure S1 [file CAM4-12-760-s002.docx]

**
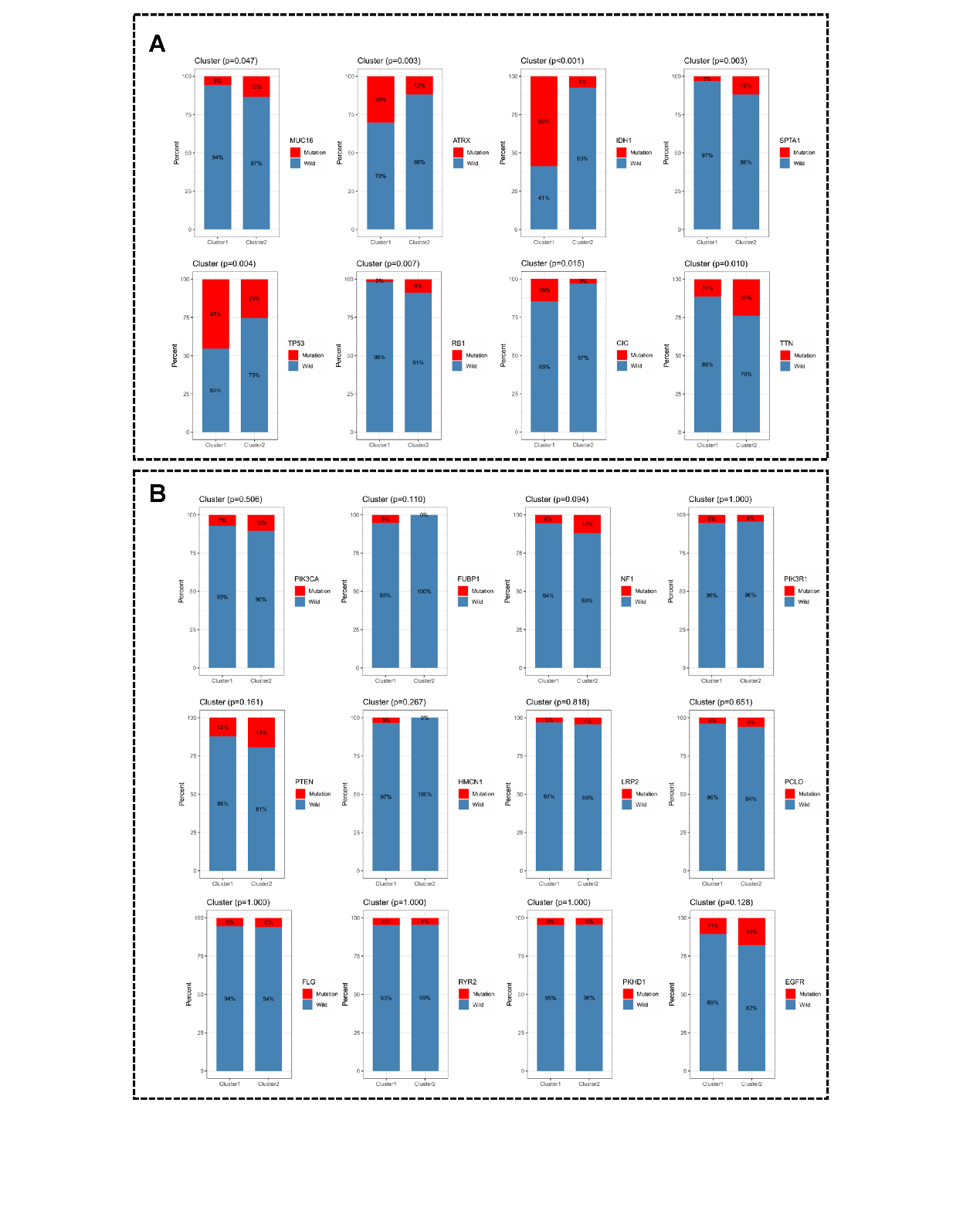
**

**Figure S1**. (**A)** Genes with the significant difference in mutation frequency between cluster1 and cluster2. Column charts respectively show the difference in mutation frequency of eight genes between cluster1 and cluster2. p < 0.05 is considered statistically significant. **(B)** Genes with no difference in mutation frequency between cluster1 and cluster2. Column charts respectively show the difference in mutation frequency of 12 genes between cluster1 and cluster2. p < 0.05 is considered statistically significant.
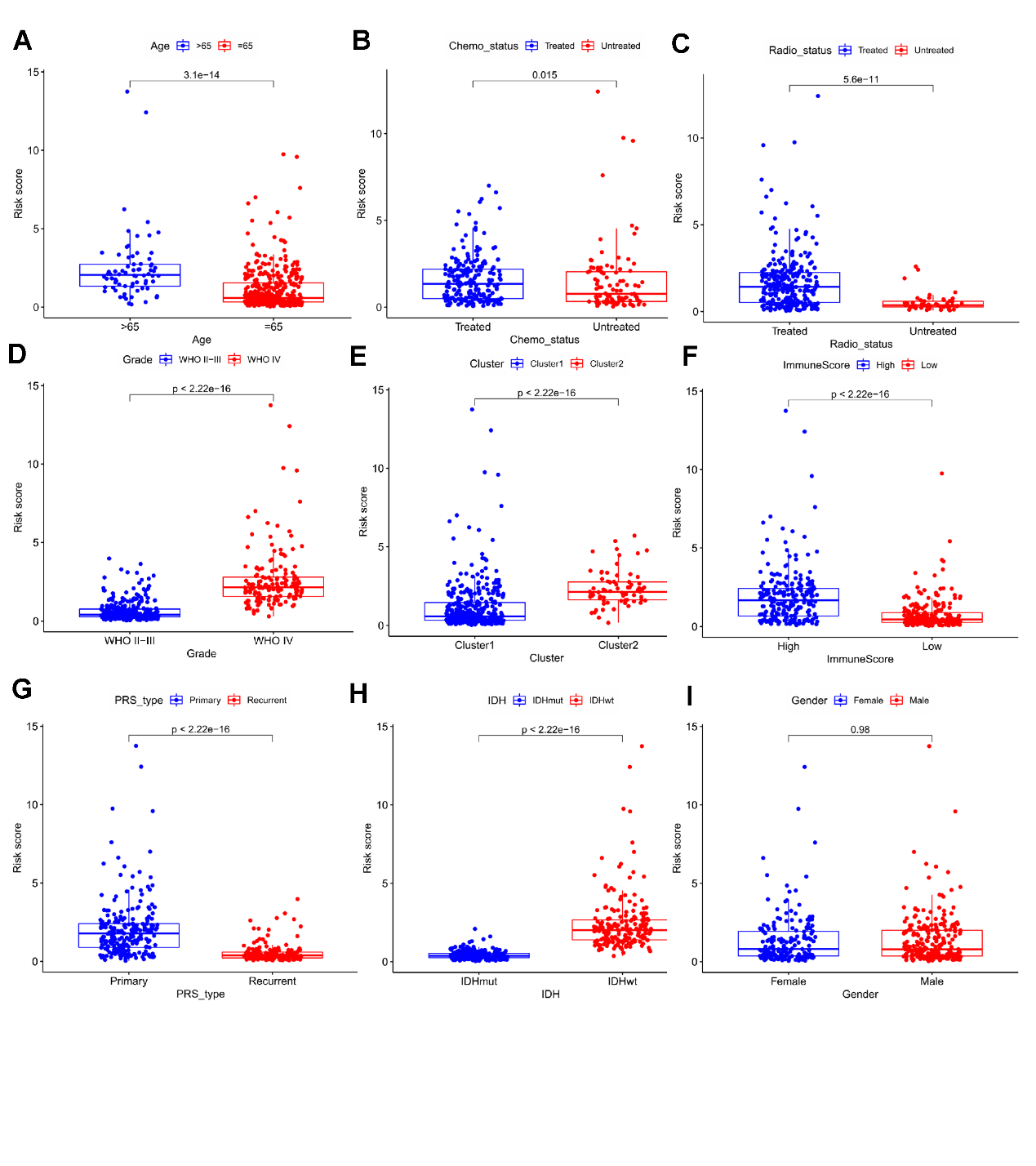


**Figure S2. (A-H)** The risk score is different in different clinicopathological subgroups. Patients with some different clinicopathological features have significantly different risk scores. These clinicopathological features include aged ≤65 or > 65 years, primary or recurrent glioma, mutant or wild-type IDH, WHO grade II-III or IV, chemotherapy treatment or untreated chemotherapy, cluster 1or cluster 2, high immune score or low immune score, radiotherapy treatment or untreated radiotherapy. p < 0.05 is considered statistically significant. (**I)** There is no significant difference between the risk scores of male patients and female patients(p = 0.98).


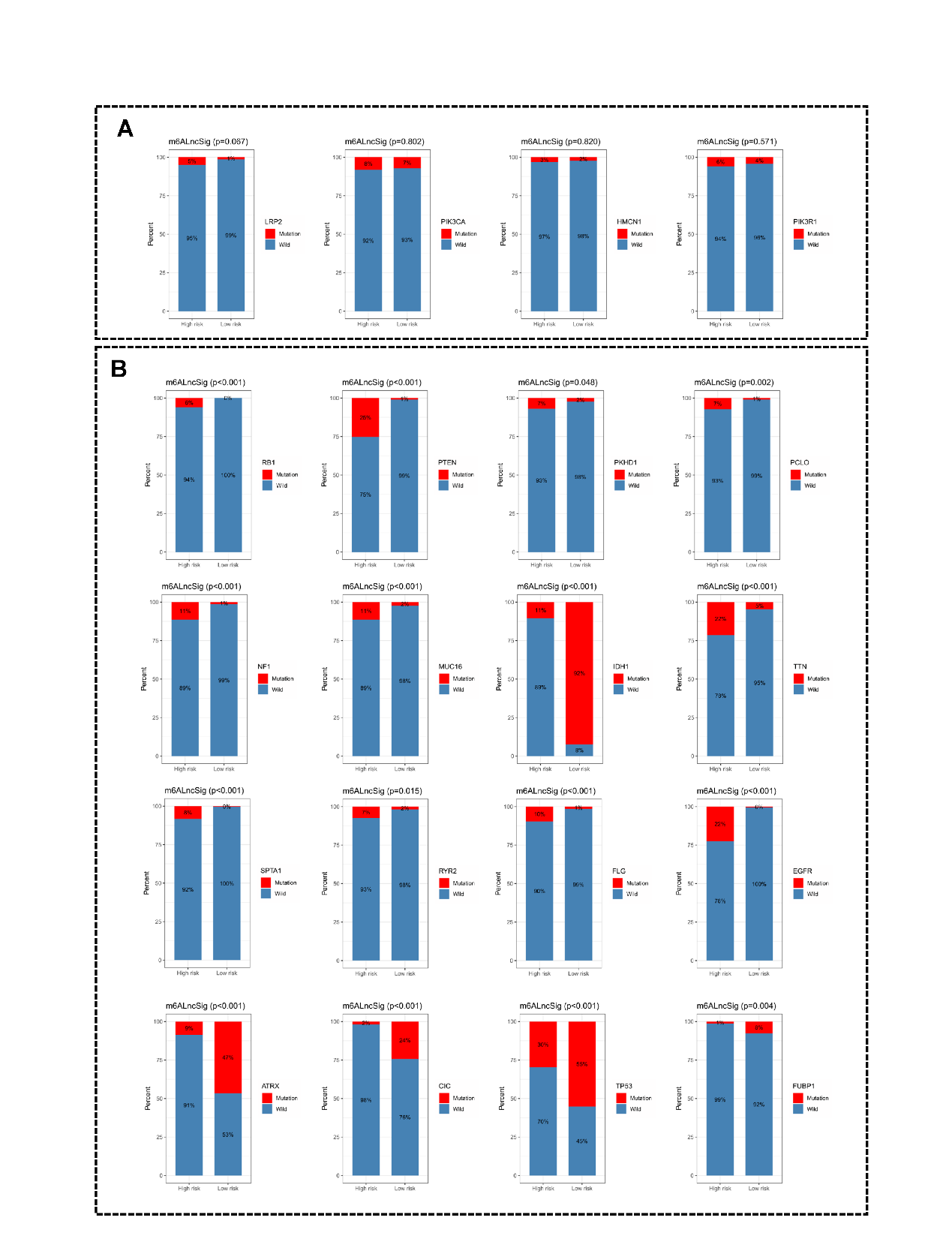


**Figure S3. (A)** Column charts respectively showed four genes whose mutation frequency showed no significant difference between the high-risk set and low-risk set. (**B)** Column charts respectively showed 16 genes whose mutation frequency showed significant difference in mutation frequency between the high-risk set and low-risk set. p < 0.05 is considered statistically significant.


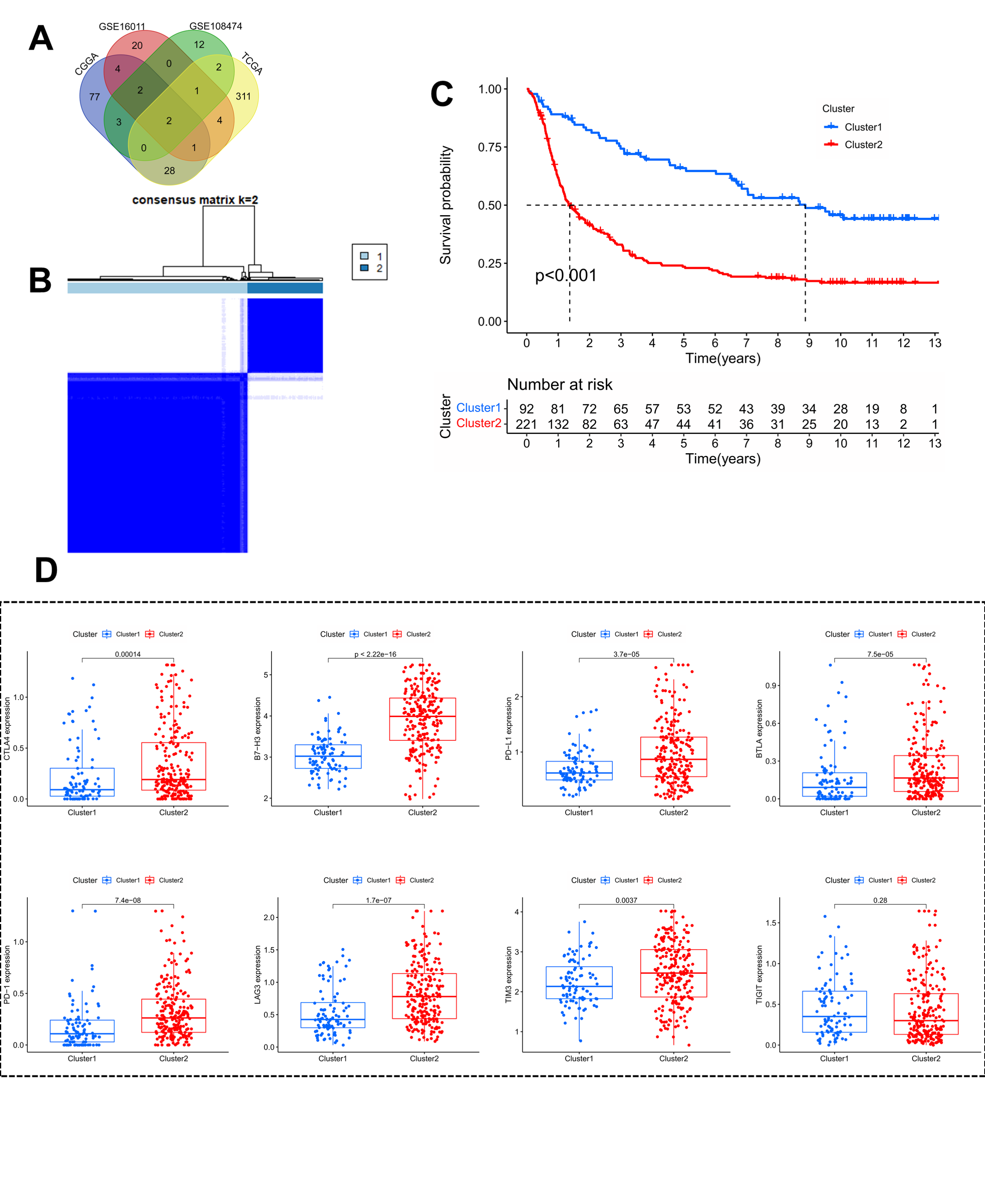


**Figure S4.** **(A)** The intersection of the m6A-related LncRNAs from four datasets (CGGA ,TCGA, GSE16011 and GSE108474). **(B)** An unsupervised clustering analysis (k-means clustering) was performed in CGGA cohort with 500 repetitions to guarantee the stability of classification. The analysis clustered the patients from CGGA into cluster1 and cluster2. **(C)** Survival analysis between patients in cluster 1 or cluster 2. Cluster 1 showed a better prognosis than cluster 2 in patients with glioma. **(D)** Comparison of immune gene expressed between cluster 1 and cluster 2. p < 0.05 is considered statistically significant.


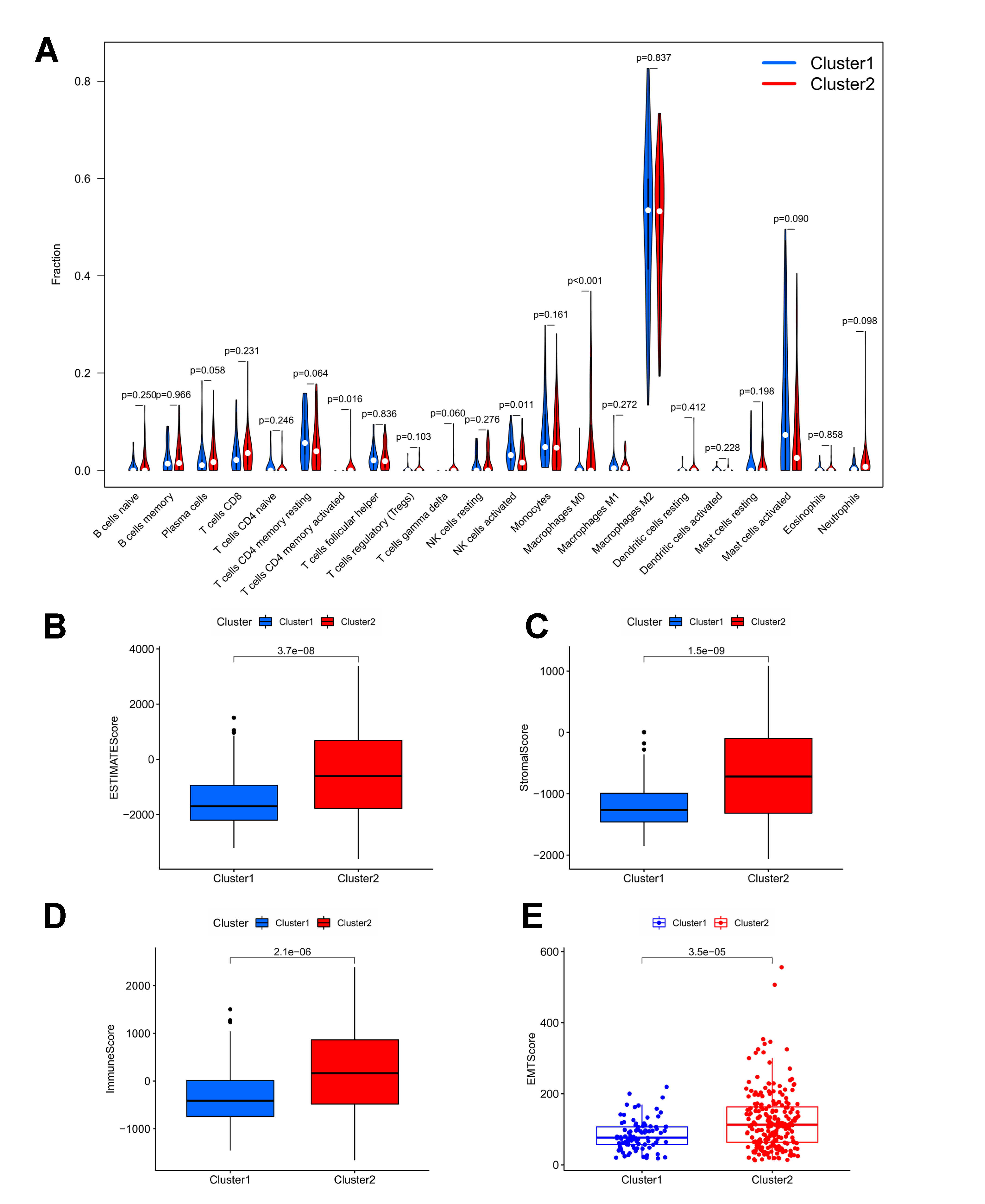


**Figure S5. (A)** The analysis of the CIBERSORT to compare the infiltration of the immune cells in CGGA cluster 1 and cluster 2. **(B)** The ESTIMATE Score between CGGA cluster 1 and cluster 2. **(C)** The Stromal Score between CGGA cluster 1 and cluster 2. **(D)** The Immune Score between CGGA cluster 1 and cluster 2. **(E)** The EMT analysis between CGGA cluster 1 and cluster 2. p < 0.05 is considered statistically significant.


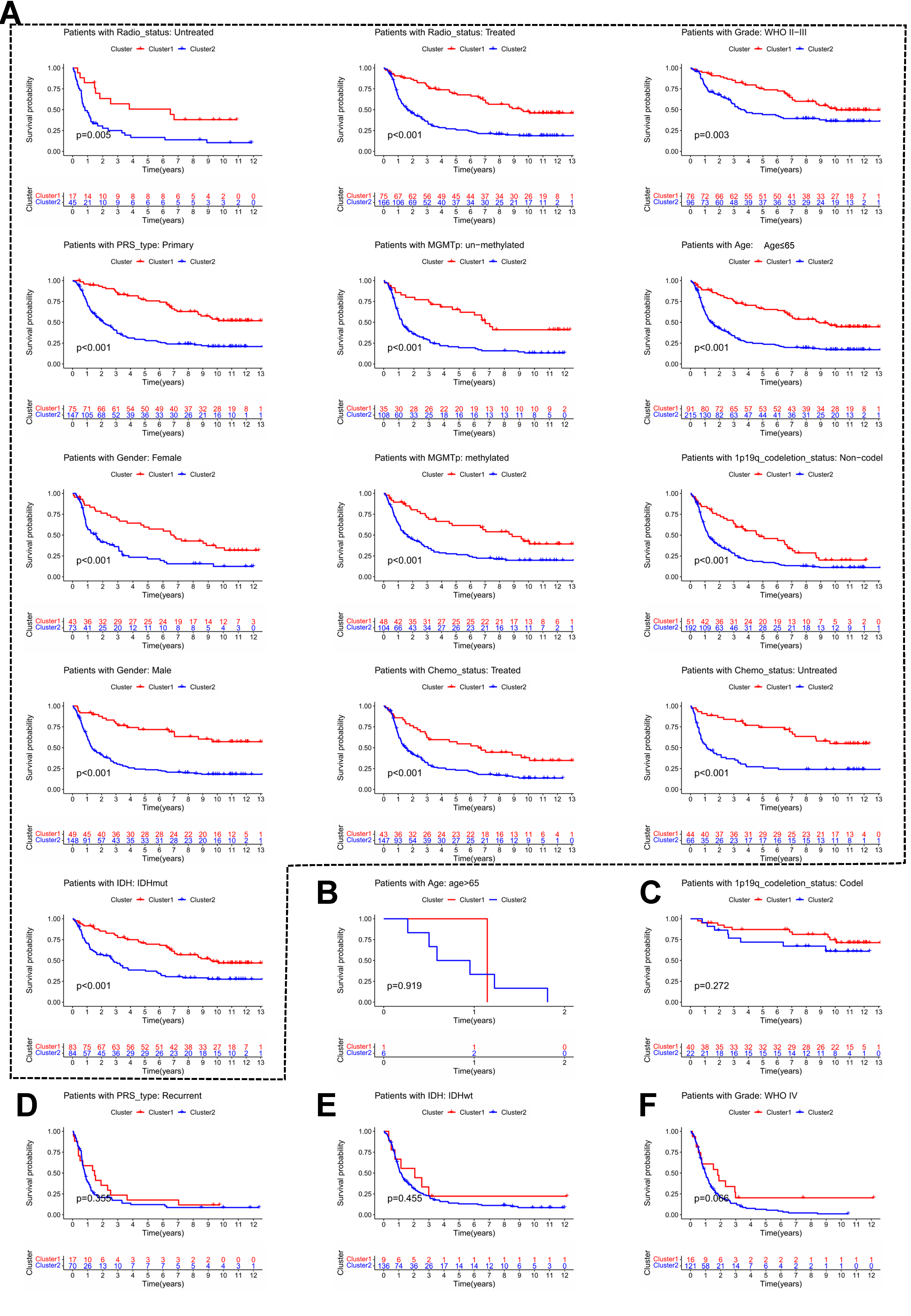


**Figure S6. (A)** The survival analysis showed that cluster 1 had a significantly better survival prognosis than cluster 2 in different clinical subgroups. **(B-F)** The survival analysis showed that there was no significant difference in survival prognosis between cluster 1 and cluster 2 in patients with age＞65, with 1p19q codeletion, with recurrent PRS, with IDHwt, or with WHO IV. p < 0.05 is considered statistically significant.


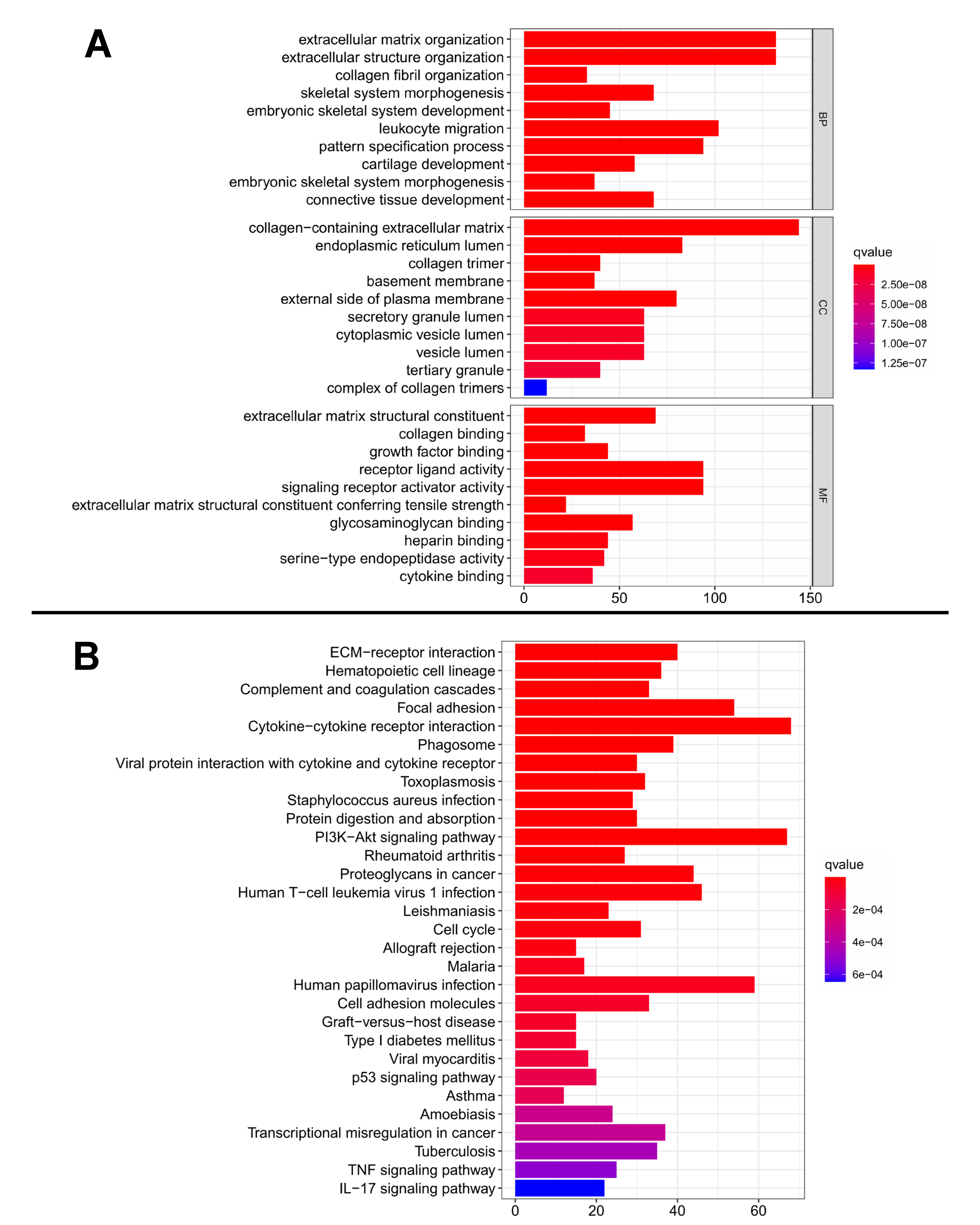


**Figure S7. (A)** GO analysis was conducted in differentially expressed genes between CGGA cluster 1 and cluster 2. **(B)** KEGG analysis was conducted in differentially expressed genes between CGGA cluster 1 and cluster 2.


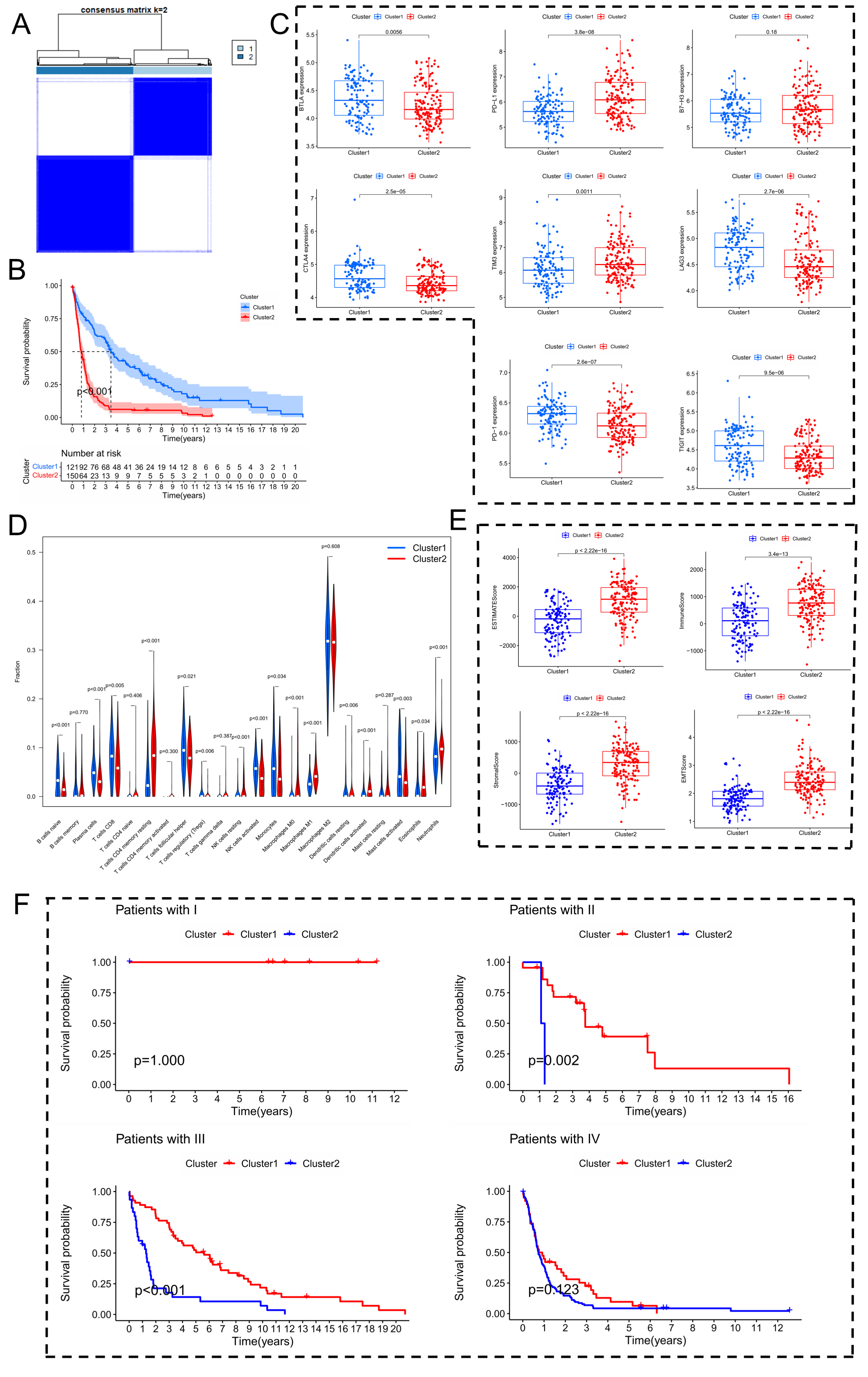


**Figure S8. Validation in dataset GSE16011 (A)** An unsupervised clustering analysis (k-means clustering) was performed in GSE16011 cohort **(B)** Survival analysis between patients in cluster 1 or cluster 2. Cluster 1 showed a better prognosis than cluster 2 in patients with glioma. **(C)** Comparison of immune gene expressed between cluster 1 and cluster 2. p < 0.05 is considered statistically significant. **(D)** The analysis of the CIBERSORT to compare the infiltration of the immune cells in GSE16011 cluster 1 and cluster 2. **(E)** The ESTIMATE Score, the Stromal Score, the immune score and the EMT analysis between GSE16011 cluster 1 and cluster 2. The p < 0.05 is considered statistically significant. **(F)** The survival analysis showed that cluster 1 had a significantly better survival prognosis than cluster 2 in patients with WHO Ⅱ and Ⅲ. For the patients with WHO Ⅰ and Ⅳ, there was no significant difference in survival prognosis between cluster 1 and cluster 2. The p < 0.05 is considered statistically significant.


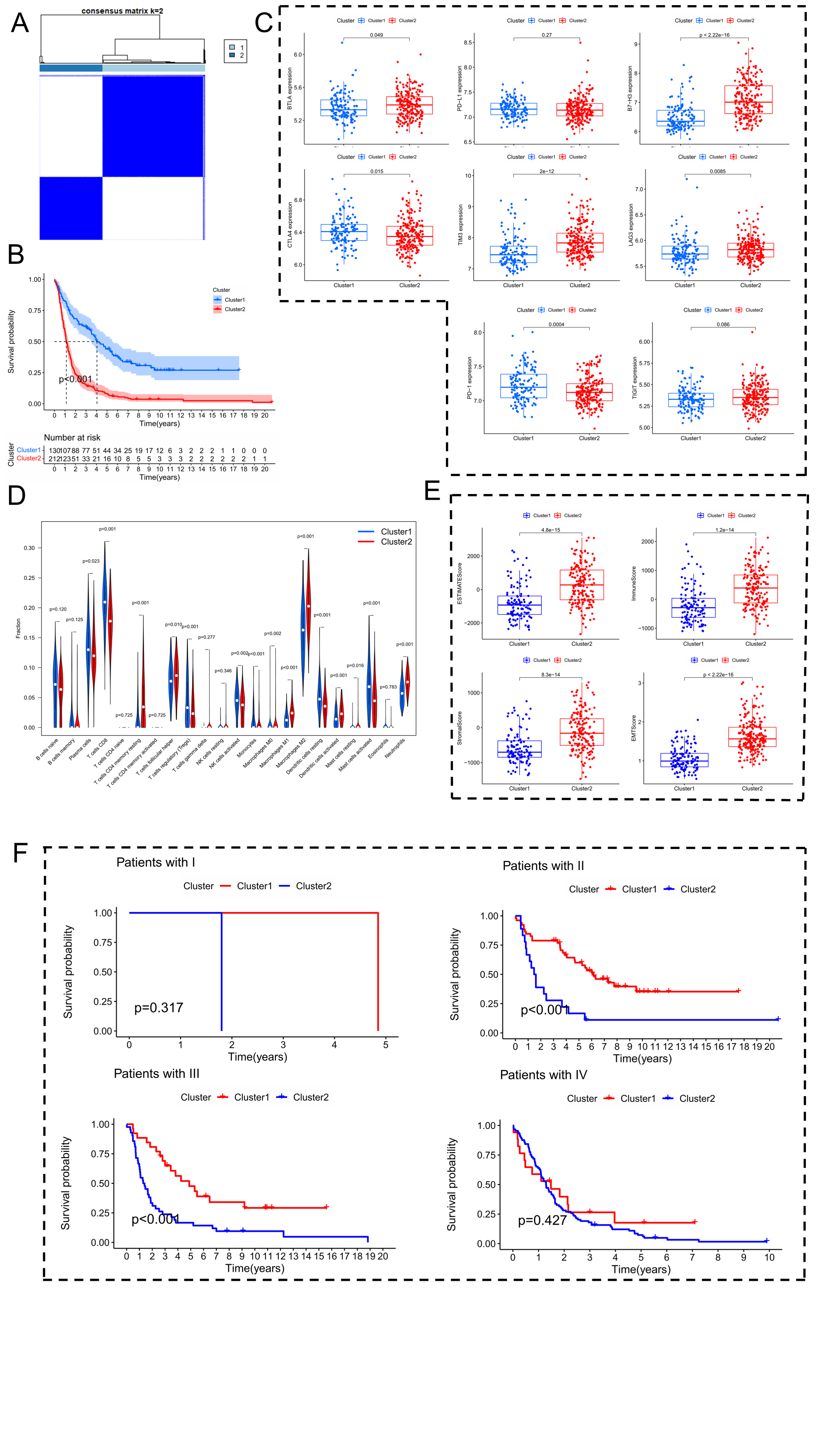


**Figure S9. Validation in dataset GSE108474 (A)** An unsupervised clustering analysis (k-means clustering) was performed in GSE108474 cohort **(B)** Survival analysis between patients in cluster 1 or cluster 2. Cluster 1 showed a better prognosis than cluster 2 in patients with glioma. **(C)** Comparison of immune gene expressed between cluster 1 and cluster 2. p < 0.05 is considered statistically significant. **(D)** The analysis of the CIBERSORT to compare the infiltration of the immune cells in GSE108474 cluster 1 and cluster 2. **(E)** The ESTIMATE Score, the Stromal Score, the immune score and the EMT analysis between GSE108474 cluster 1 and cluster 2. The p < 0.05 is considered statistically significant. **(F)** The survival analysis showed that cluster 1 had a significantly better survival prognosis than cluster 2 in patients with WHO Ⅱ and Ⅲ. For the patients with WHO Ⅰ and Ⅳ, there was no significant difference in survival prognosis between cluster 1 and cluster 2. The p < 0.05 is considered statistically significant.
